# Exercise Snacks and Acute Postprandial Glucose and Insulin Metabolism: An Umbrella Review and De Novo Meta-Analysis

**DOI:** 10.3390/metabo16090635

**Published:** 2026-08-31

**Authors:** Chuyuan Qiao, Chuwei Yang, Shenglei Yang, Yan Wang

**Affiliations:** School of Sports Medicine and Rehabilitation, Beijing Sport University, Beijing 100084, China; xiaosadashuai@163.com (C.Q.); chuizigou@163.com (C.Y.); y17616251939@163.com (S.Y.)

**Keywords:** exercise snacks, postprandial metabolism, glucose metabolism, insulin response, metabolic regulation, umbrella review, meta-analysis

## Abstract

**Background/Objectives:** Exercise snacks—brief, purposeful bouts of physical activity distributed throughout the day—may modify postprandial metabolic regulation, particularly glucose handling and insulin responses, while reducing time barriers to structured exercise. However, repeated primary studies and variable definitions may overstate evidence maturity. We reappraised evidence on exercise snacks and acute postprandial metabolism using review-level evaluation and independent study families. **Methods:** We conducted an umbrella review with de novo meta-analyses when evidence was sufficiently comparable. Six bibliographic databases and three supplementary literature-discovery sources were searched through 5 August 2026. Exercise snacks were defined as active bouts ≤ 15 min, performed at least twice per intervention day, and distributed across the day. AMSTAR 2, ROBIS, and corrected covered area (CCA) assessed review quality, risk of bias, and overlap. Random-effects models used restricted maximum likelihood with Hartung–Knapp adjustment; paired crossover effects were Hedges’ *g_z_*. RoB 2 and GRADE were applied. **Results:** Twenty-one systematic reviews were included; outcome-specific CCA ranged from 11.2% to 30.2%. After deduplication, only four study families contributed to acute plasma glucose iAUC, three to plasma insulin iAUC, and two to exploratory acute CGM glucose iAUC. Given these small evidence sets and the conservative Hartung–Knapp adjustment, the analyses had limited precision and statistical power to detect small-to-moderate effects. No statistically clear pooled effect was observed for plasma glucose (Hedges’ *g_z_* = −0.127, 95% CI −1.111 to 0.857; *I*^2^ = 77.9%) or insulin iAUC (Hedges’ *g_z_* = −0.520, 95% CI −1.263 to 0.222; *I*^2^ = 15.3%). The two-study CGM synthesis was descriptive and hypothesis-generating and neither supported nor refuted an acute glucose effect. **Conclusions:** Review-level abundance exceeded the independent quantitative evidence available for robust inference. The current analyses were underpowered to establish small-to-moderate pooled effects of exercise snacks on acute postprandial glucose or insulin metabolism; therefore, the findings indicate limited and statistically unstable evidence rather than evidence of no metabolic benefit.

## 1. Introduction

The World Health Organization 2020 guidelines on physical activity and sedentary behavior recommend that adults limit sedentary time and replace it with physical activity of any intensity whenever possible [1]. A harmonized meta-analysis of accelerometer data likewise showed that higher physical activity and lower sedentary time were associated with reduced all-cause mortality [2]. Yet guideline recommendations do not automatically translate into real-world feasibility: time constraints, low physical fitness, and work- or health-related burdens may limit sustained, structured exercise. Exercise snacks have therefore attracted interest as a strategy that breaks exercise into shorter bouts that can be more readily embedded in daily life, thereby reducing the time demand of each exercise session [3].

The literature on brief, distributed activity includes related but non-equivalent concepts such as exercise snacks, activity breaks, short bouts of accumulated exercise, and active interruptions of prolonged sitting. Francois et al. [4] were among the first to use the term “exercise snacks” for brief high-intensity activity performed before meals, and the concept was later extended to more accessible formats such as intermittent stair climbing [5]. Vigorous intermittent lifestyle physical activity (VILPA), by contrast, emphasizes incidental short bursts of vigorous activity arising in daily life [6], while a recent consensus further characterized short bouts of accumulated exercise by bout duration, daily frequency, and recovery interval [7]. These approaches share an emphasis on brief, distributed activity but differ in exercise intensity, behavioral context, and prescription structure.

These conceptual differences directly affect evidence synthesis. Existing systematic reviews have separately examined the acute metabolic effects of actively interrupting prolonged sitting [8,9,10] and exercise snacks under stricter definitions [11], with substantial variation in eligibility criteria, outcome constructs, time windows, and definitions of brief activity. Moreover, the same primary studies may be repeatedly included across reviews. Corrected covered area (CCA) can quantify such overlap [12]; if overlap is ignored, growth in the number of reviews may be mistaken for growth in independent evidence and may overstate the apparent maturity of the evidence base.

Primary trials also suggest that metabolic responses to brief, distributed activity are highly protocol- and context-dependent. Dunstan et al. [13] and Peddie et al. [14] observed improvements in postprandial glucose and/or insulin responses when prolonged sitting was interrupted with brief activity in adults with overweight/obesity and in healthy adults, respectively. Similar findings have been reported in adults with type 2 diabetes, with some metabolic improvements persisting overnight [15,16]. However, not all metabolic outcomes change in parallel: simple resistance activities reduced postprandial insulin responses but did not clearly alter glucose iAUC in adults with overweight/obesity [17]. Post-meal walking, meal–exercise timing, and the glycemic characteristics of the test meal may further modify acute metabolic responses [18,19,20].

Given these inconsistencies, we did not use the number of reviews or isolated favorable findings as a proxy for evidence maturity. Instead, we applied a two-level evidence-reappraisal framework. Within this framework, acute plasma glucose and insulin iAUC were treated as the principal quantitative markers of postprandial metabolic response. At the review level, we assessed methodological quality, risk of bias, and primary-study overlap and mapped the scope of the evidence. We then traced eligible evidence back to primary studies and used independent study families, rather than publication counts, as the unit of quantitative evidence. De novo meta-analysis was undertaken only when populations, interventions, comparators, outcomes, time windows, and effect scales were sufficiently comparable. This framework was designed to distinguish the apparent volume of the review literature from the amount of independent primary evidence available for robust inference on exercise-induced metabolic responses.

## 2. Materials and Methods

We conducted an umbrella review and, where primary evidence was sufficiently comparable, de novo meta-analyses at the level of independent study families. The protocol was prospectively registered in PROSPERO (CRD420261460040), and reporting followed PRIOR, PRISMA 2020, and PRISMA-S [21,22,23]. Post-registration methodological refinements addressed the operational definition of exercise snacks, the handling of reports for which full text could not be obtained, and the addition of acute CGM glucose iAUC as an exploratory outcome. These refinements were updated in the PROSPERO record and are transparently documented in Supplementary Table S9. Because only aggregate data from published studies were used, no additional ethical approval or individual informed consent was required.

### 2.1. Eligibility Criteria

Eligibility criteria were structured according to the PICOS framework. Population: adults aged ≥ 18 years. Intervention: exercise snacks or related active interruptions of prolonged sitting. Comparator: uninterrupted sitting, usual activity/no intervention, continuous exercise, alternative eligible exercise-snack doses or modes, or other active comparators. Outcomes: cardiometabolic outcomes, physical fitness, physical function, and body composition. Study design: systematic reviews, with or without meta-analysis, that used a systematic search and study-selection process and included human intervention studies. Narrative reviews, scoping reviews, protocols, conference-only records, and reports that did not meet the prespecified review-design criteria were excluded.

For the de novo component, exercise snacks were operationally defined as purposeful bouts of active physical activity lasting ≤ 15 min per bout, performed at least twice per intervention day, and distributed across the day rather than concentrated into a single conventional exercise session. This operational definition was informed by established exercise-snack concepts and the recent consensus on short bouts of accumulated exercise [3]. Standing-only interruptions, bouts > 15 min, and interventions performed only once per day were excluded. Successive bouts were required to be clearly discrete and distributed across the day rather than concentrated into a single conventional exercise session. No fixed minimum inter-bout interval was imposed beyond this requirement. Eligible modes included walking, stair climbing, resistance exercise, sit-to-stand exercise, cycling, and other purposeful active movement. The full operational definition and eligibility framework are provided in Supplementary Table S1.

We distinguished prescribed exercise snacks from active interruptions of prolonged sitting whose primary purpose was to break up sedentary time. The latter were eligible for the de novo component only when they met the same requirements for active movement, bout duration, daily frequency, and within-day distribution. If a systematic review included both eligible and ineligible interventions, eligibility was reassessed at the primary-study or intervention-arm level rather than excluding the review solely because it used a broader intervention definition.

For primary studies entering the de novo synthesis, the relevant PICOS criteria had to be satisfied at the study or intervention-arm level; the original report also had to be identifiable, and the effect estimate and its variance had to be extractable or reliably derivable.

### 2.2. Search Strategy

We searched six bibliographic databases—PubMed/MEDLINE, Embase, Web of Science Core Collection, Scopus, the Cochrane Database of Systematic Reviews, and SPORTDiscus—and three supplementary literature-discovery sources (Europe PMC, OpenAlex, and Crossref) through 5 August 2026. All records were combined and deduplicated before screening. Search terms covered exercise snacks and adjacent concepts, including accumulated exercise, brief exercise bouts, activity breaks, and breaking up prolonged sitting, combined with terms for systematic reviews or meta-analyses. Population, disease, age, and outcome terms were not mandatory search concepts to reduce the risk of missing earlier studies or reports using different terminology.

The search strategy was intentionally broader than the final operational definition. Requirements for bout duration, daily frequency, active movement, and other intervention characteristics were applied during eligibility assessment rather than through restrictive search terms. The complete source-specific search strategies and query syntax are reproduced in Supplementary File S1. Citation tracking and screening records were retained in the review audit trail, and the final screening set was locked on 7 August 2026.

### 2.3. Study Selection

Search records were deduplicated primarily by DOI, with additional manual verification using PMID, title, author, and publication year. Two reviewers (CQ and CY) independently screened titles/abstracts and assessed full-text eligibility. Disagreements were resolved by discussion, with adjudication by a third reviewer when necessary. Under the locked retrieval rule, reports for which full text could not be obtained were classified as not retrieved and did not proceed to full-text eligibility assessment.

### 2.4. Data Extraction and Study-Family Construction

Candidate primary studies for the de novo component were identified mainly from the included systematic reviews and, where applicable, supplemented by records identified in the final search update. Relationships among reports were determined using DOI, author–year combinations, trial or registration identifiers, participant and setting characteristics, intervention details, and other cohort-level information. Multiple reports arising from the same underlying cohort or experiment were grouped into a single original-study family; the study family, rather than publication count, served as the unit of statistical independence to avoid double counting.

To reduce the risk of missing eligible primary studies, we examined the reference lists and primary-study inventories of all included reviews; reconciled cited reports using DOI, author–year information, trial identifiers, and cohort characteristics; and screened potentially relevant records identified during the final search update. Nevertheless, because the database search strategy was designed primarily to identify systematic reviews rather than all individual trials, these procedures could not guarantee retrieval of an eligible primary study that had not been cited by any included review.

Structured data extraction covered study-family membership, population and design, comparator, activity mode and dose, bout duration and frequency, intervention timing, outcome definition, measurement medium, time window, effect estimate, variance information, data source, and verification status. A second reviewer verified key eligibility and quantitative fields; information that could not be verified or reliably reconstructed was excluded from formal quantitative synthesis. Study-family effect estimates and variance sources for the main paired Hedges’ *g_z_* analyses are reported in Supplementary Table S2. For primary-study families contributing to the acute metabolic synthesis, we additionally extracted the reported test-meal energy and macronutrient composition, meal timing, timing of the first eligible activity bout relative to meal consumption, subsequent inter-bout intervals, and the metabolic outcome integration window.

When published summary statistics permitted a reliable statistical conversion, required parameters were derived using prespecified formulas from standard errors, confidence intervals, or other summary statistics. Otherwise, the outcome was retained for narrative synthesis. When published aggregate information remained insufficient, we did not request individual participant data. For Gale 2023 [24], we contacted the investigators to request the aggregate paired variance information required for effect-scale conversion, but no response had been received by the time of revision. No individual participant data were imputed.

### 2.5. Metabolic Outcomes and Time Windows

At registration, the planned outcome domains comprised glucose and insulin regulation as the principal metabolic domain, with blood lipids, blood pressure, physical fitness/physical function, and body composition as additional domains. The main quantitative metabolic outcomes were acute plasma glucose iAUC and acute plasma insulin iAUC, analyzed separately as markers of postprandial metabolic response. Incremental AUC, total AUC, peak concentration, and mean concentration were treated as distinct outcome constructs. Acute CGM glucose iAUC was not prespecified as a separate measurement-medium outcome. It was added after registration as a supplementary exploratory outcome because comparable acute study-level data became available. This refinement did not alter the eligibility or inclusion of the 21 systematic reviews. At the primary-study level, it only allowed for two otherwise eligible study families to be allocated to a separate exploratory CGM synthesis. CGM-derived glucose outcomes were analyzed separately from plasma glucose, and longer-term CGM outcomes remained outside the formal quantitative synthesis.

Secondary outcome domains included blood lipids, blood pressure, physical fitness/physical function, and body composition. Individual lipid fractions, systolic and diastolic blood pressure, different fitness/function tests, and distinct body-composition measures were treated separately. A de novo meta-analysis was performed only when at least two independent study families were sufficiently comparable in population, intervention, comparator, outcome definition, measurement method, time window, and effect scale. Time windows were prespecified as acute (<24 h), transitional (24 h to <2 weeks), short- to medium-term (≥2 weeks to <6 months), and long-term (≥6 months); outcomes from different time scales were not pooled. This categorization was used to distinguish acute experimental responses from longer-duration training exposure and was informed by the acute-versus-longer-term distinction used in the exercise-snack literature [7,11].

### 2.6. Quality, Risk-of-Bias, and Evidence-Overlap Assessment

AMSTAR 2 was used to appraise the methodological quality of included systematic reviews, and ROBIS was used to assess review-level risk of bias [25,26]. Two reviewers performed assessments independently and resolved disagreements by consensus. All eligible reviews were retained in the structured umbrella-review synthesis; quality and risk-of-bias judgments informed interpretation of credibility rather than acting as simple inclusion or exclusion thresholds.

To quantify overlap of primary evidence across reviews, we constructed a citation matrix at the study-family level and calculated outcome-specific CCA. CCA was used only to describe the extent of shared primary evidence and to support evidence-source allocation and study-family deduplication; it was not interpreted as a measure of intervention effect. Outcome-specific CCA results are reported in Supplementary Table S6 [12].

For primary studies contributing to de novo quantitative analyses, risk of bias was assessed with RoB 2 at the study-family–outcome level [27], using the crossover-trial approach where applicable. GRADE was used to assess certainty for each outcome-specific evidence body [28]. Detailed RoB 2 judgments are provided in Supplementary Table S4 and visualized in Supplementary Figure S4, while detailed GRADE assessments are presented in Supplementary Table S5. Because each evidence body contained fewer than 10 independent study families, funnel-plot asymmetry and small-study effects were not formally assessed.

### 2.7. Effect Measures and Data Conversion

For continuous outcomes measured on the same scale, mean differences were preferred; when outcomes were conceptually comparable but measured on different scales, standardized mean differences were considered. Paired continuous outcomes from crossover trials entering the main de novo analyses were expressed as Hedges’ *g_z_*. Effect direction was harmonized such that, where clinically meaningful, negative values indicated lower outcome values under the exercise-snack condition than under the comparator. Results reported on fundamentally different effect scales, such as log-ratios, were not pooled with Hedges’ *g_z_*; non-pooled single-study results are reported in Supplementary Table S3.

Where necessary, analysis parameters were derived from standard errors, confidence intervals, or *p* values. When the within-participant variance required for paired crossover comparisons was unavailable, the primary analysis assumed a within-participant correlation of *r* = 0.50, with sensitivity analyses at *r* = 0.25 and *r* = 0.75. For multi-arm studies, shared comparators were not counted more than once. When multiple eligible intervention arms could not be appropriately combined, one prespecified eligible arm was included in the primary analysis and alternative eligible arms were examined in sensitivity analyses.

### 2.8. Data Synthesis and Statistical Analysis

At the review level, eligible evidence was synthesized using a structured narrative approach. For primary-study evidence, de novo meta-analysis was undertaken only when at least two independent study families showed sufficient clinical and methodological comparability in population, intervention mode and dose, comparator, outcome construct, measurement medium, time window, and effect scale. Outcomes that did not meet these criteria remained in the structured review-level synthesis. Pooling decisions were based primarily on clinical and methodological comparability rather than on statistical heterogeneity thresholds alone.

The main quantitative analyses used random-effects models. Between-study variance was estimated by restricted maximum likelihood (REML), with Hartung–Knapp adjustment of 95% confidence intervals when estimable [29,30]. In addition to pooled effect estimates and 95% confidence intervals, we reported the number of independent study families and characterized heterogeneity using *τ*^2^, *I*^2^, and Cochran’s *Q*; 95% prediction intervals were reported when they added meaningful information [31]. Because these statistics describe different aspects of heterogeneity, we did not classify heterogeneity mechanically using a single *I*^2^ threshold.

For crossover trials lacking a reported within-participant correlation, the primary analysis assumed *r* = 0.50; sensitivity analyses used *r* = 0.25 and *r* = 0.75. Additional sensitivity analyses used alternative eligible intervention arms, leave-one-out analyses, and fixed-effect models; fixed-effect estimates were used only to assess robustness and did not replace interpretation of the prespecified random-effects models. Prespecified potential sources of heterogeneity included participant health or metabolic-risk status, activity mode and intensity, bout duration, daily frequency, activity timing, and intervention duration. Subgroup analyses or meta-regression were considered only when the number of independent studies was sufficient. Full sensitivity analyses are reported in Supplementary Table S8 and Supplementary Figures S2 and S3.

The value *r* = 0.50 was selected as a moderate midpoint assumption when no empirical within-participant correlation was reported, thereby avoiding either an independence assumption (*r* = 0) or an implausibly strong pairing assumption. The alternative values of *r* = 0.25 and *r* = 0.75 represented lower and higher within-participant dependence and were used to evaluate the sensitivity of the estimated standard errors, study weights, and pooled confidence intervals to this unobserved parameter. The assumed correlation affected the variance of the paired effect estimate but not the observed direction of the within-study mean difference.

Because each quantitative evidence body contained fewer than 10 independent study families, funnel plots and related statistical tests were not used formally to assess small-study effects or publication bias; absence of such testing was not interpreted as absence of publication bias. All quantitative analyses were conducted in R (version 4.6.1), primarily using the meta and metafor packages [32,33]. All tests were two-sided; 95% confidence intervals were reported, and the nominal significance level was *α* = 0.05. Interpretation integrated effect estimates and their uncertainty, heterogeneity, sensitivity analyses, and GRADE certainty rather than statistical significance alone.

## 3. Results

### 3.1. Literature Search

**Figure 1:**
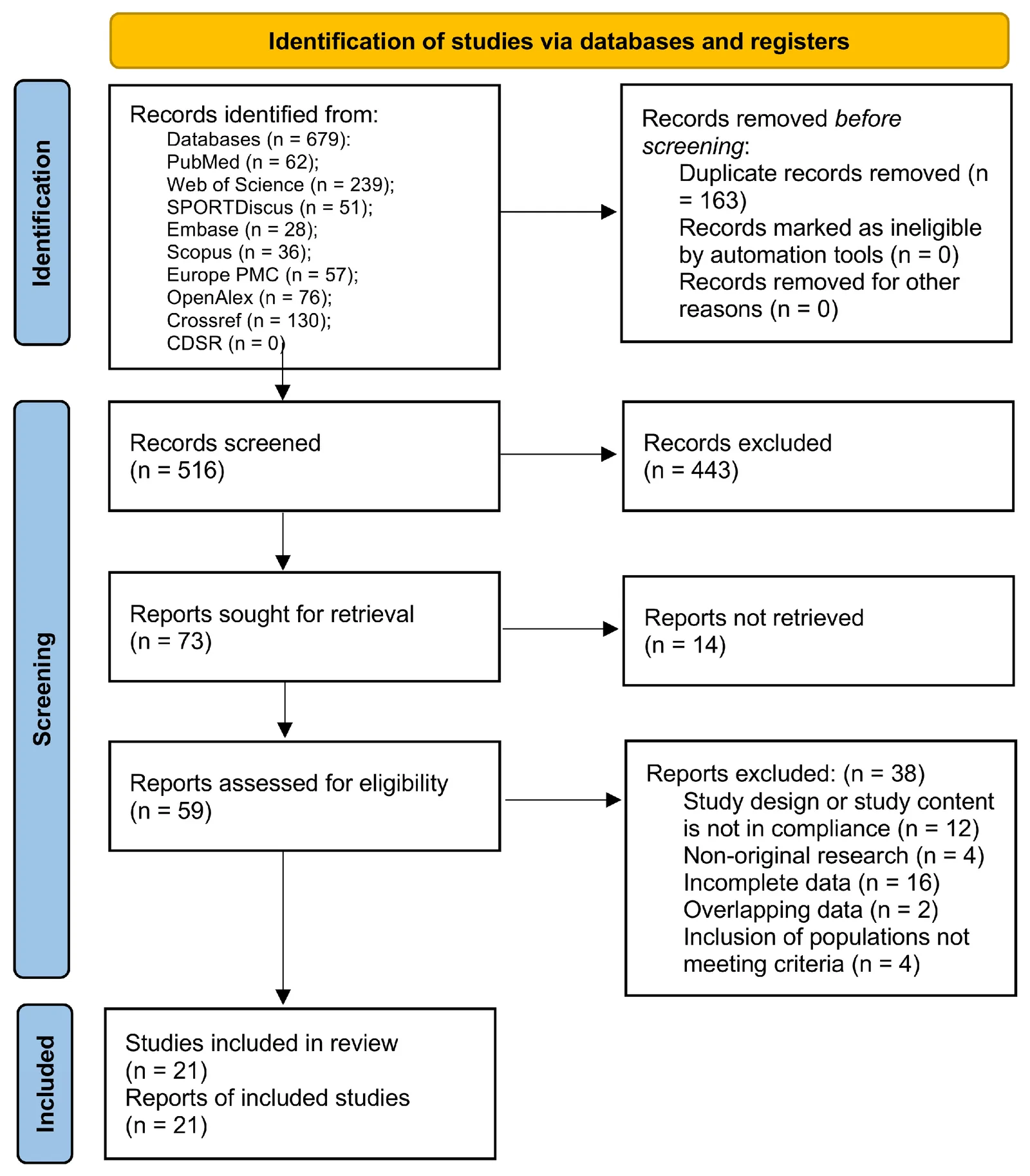
PRISMA flow diagram of study selection.

The search identified 679 records, of which 163 duplicates were removed. The remaining 516 records underwent title/abstract screening, and 443 were excluded. Seventy-three reports were sought for retrieval, of which 14 were not retrieved. The remaining 59 reports underwent full-text eligibility assessment; 38 were excluded, leaving 21 systematic reviews for inclusion (Figure 1). The final database search was completed on 5 August 2026, and screening was locked on 7 August 2026.

### 3.2. Review Characteristics and Quality Appraisal

The 21 included systematic reviews were published between 2018 and 2026 and addressed exercise snacks, accumulated short-bout exercise, and/or active interruptions of prolonged sitting. Populations included generally healthy or physically inactive adults, adults with overweight/obesity or elevated metabolic risk, and older adults. Outcomes spanned acute glucose and insulin responses, other cardiometabolic markers, physical fitness/physical function, and body composition (Table 1) [8,9,10,11,34,35,36,37,38,39,40,41,42,43,44,45,46,47,48,49,50]. Because terminology and intervention criteria differed across reviews, all candidate primary studies were reassessed for eligibility at the study-family and intervention levels according to our operational definition before consideration for quantitative synthesis.

After study-family deduplication, four independent study families contributed to the plasma glucose iAUC analysis, three to the plasma insulin iAUC analysis, and two to the exploratory acute CGM glucose iAUC analysis. Because the same study family could contribute to more than one outcome, these counts should not be summed. Study-family effect estimates for the main quantitative pools are provided in Supplementary Table S2. Across the seven independent study families contributing to the pooled or single-study acute metabolic evidence, the prescribed or nominal interval between successive activity bouts had a median of 30 min (range 20–45 min). Six laboratory protocols used fixed intervals of 20, 30, or 45 min, whereas Bailey 2022 [51] used a free-living prescription requiring interruptions at least every 30 min; all protocols therefore represented recurrent discrete bouts rather than a single clustered exercise session. Sex composition also varied substantially across these study families. Gao 2024 [52] and Wongpipit 2021b [53] included only men, whereas Maylor 2019 [54] included only women; the remaining mixed-sex study samples ranged from approximately balanced to predominantly female (Supplementary Table S11). These sources of attrition could not be expressed as three mutually exclusive proportions because they operated on different units and denominators. Quantitatively, deduplication reduced 122 glucose/insulin study occurrences to 73 unique families (49 repeated occurrences removed). In the newly included Zhuang 2026 [46] review, application of the operational definition excluded 4 of 23 independent cohorts—2 standing-only protocols, 1 with a 20 min bout, and 1 with only 1 bout/day—leaving 19. In the locked acute iAUC dataset, seven accessible, eligible families provided quantitative data: six contributed compatible paired Hedges’ *g_z_* estimates to at least one pool, whereas Gale 2023 [24] was not pooled because its log-ratio scale was incompatible. Thus, overlap, intervention eligibility, and outcome/effect-scale compatibility made separate, non-additive contributions to the final evidence set.

**Figure 2:**
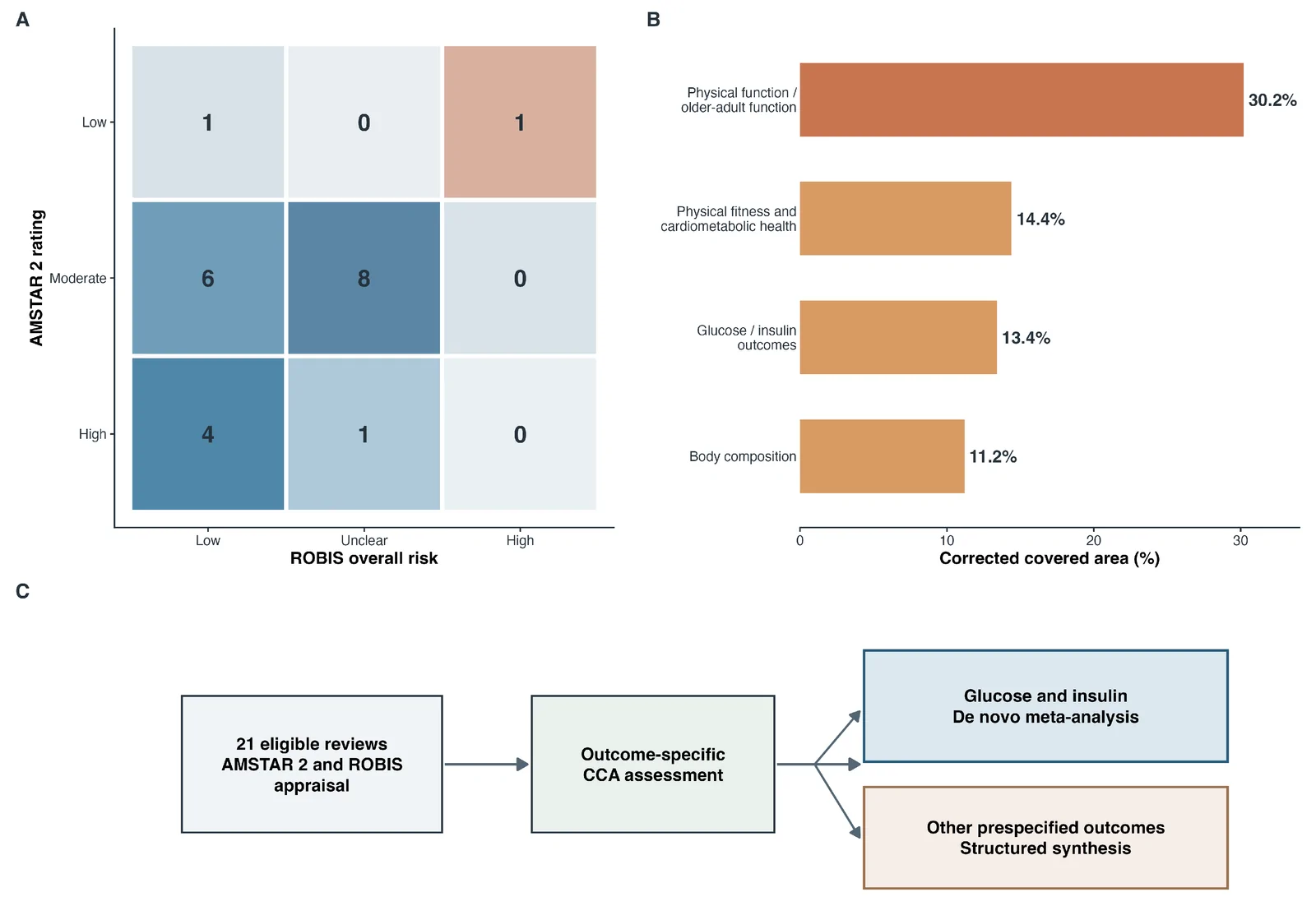
Methodological quality, risk of bias, evidence overlap, and evidence-synthesis pathway of the included systematic reviews. (**A**) Cross-tabulation of AMSTAR 2 rating and overall ROBIS risk; (**B**) outcome-specific corrected covered area; (**C**) pathway from review appraisal and overlap assessment to de novo meta-analysis or structured review-level synthesis. In panel A, darker blue shading indicates larger cell counts, whereas the peach-colored cell highlights the AMSTAR 2 low/ROBIS high-risk combination. In panel B, darker orange shading denotes a higher CCA value. Colors in panel C distinguish the synthesis pathways and do not represent quantitative effect sizes.

AMSTAR 2 rated 5 reviews as high quality, 14 as moderate quality, and 2 as low quality. ROBIS classified 11 reviews as low risk of bias, 9 as unclear risk, and 1 as high risk (Figure 2A).

Outcome-specific CCA ranged from 11.2% to 30.2%: 30.2% for physical function/older-adult function, 14.4% for physical fitness and cardiometabolic health, 13.4% for glucose and insulin, and 11.2% for body composition (Figure 2B). CCA quantified overlap only; it was used to support evidence-source allocation and study-family deduplication and was not interpreted as an effect measure. The evidence-synthesis pathway is shown in Figure 2C, with detailed CCA results in Supplementary Table S6.

### 3.3. Main Results

#### 3.3.1. Acute Plasma Glucose iAUC

Four independent study families contributed to the random-effects meta-analysis of acute plasma glucose iAUC. The pooled Hedges’ *g_z_* was −0.127 (95% CI −1.111 to 0.857; *p* = 0.709), with *τ*^2^ = 0.290, *I*^2^ = 77.9% (*Q*-test *p* = 0.004), and a 95% prediction interval of −2.103 to 1.849.

**Figure 3:**
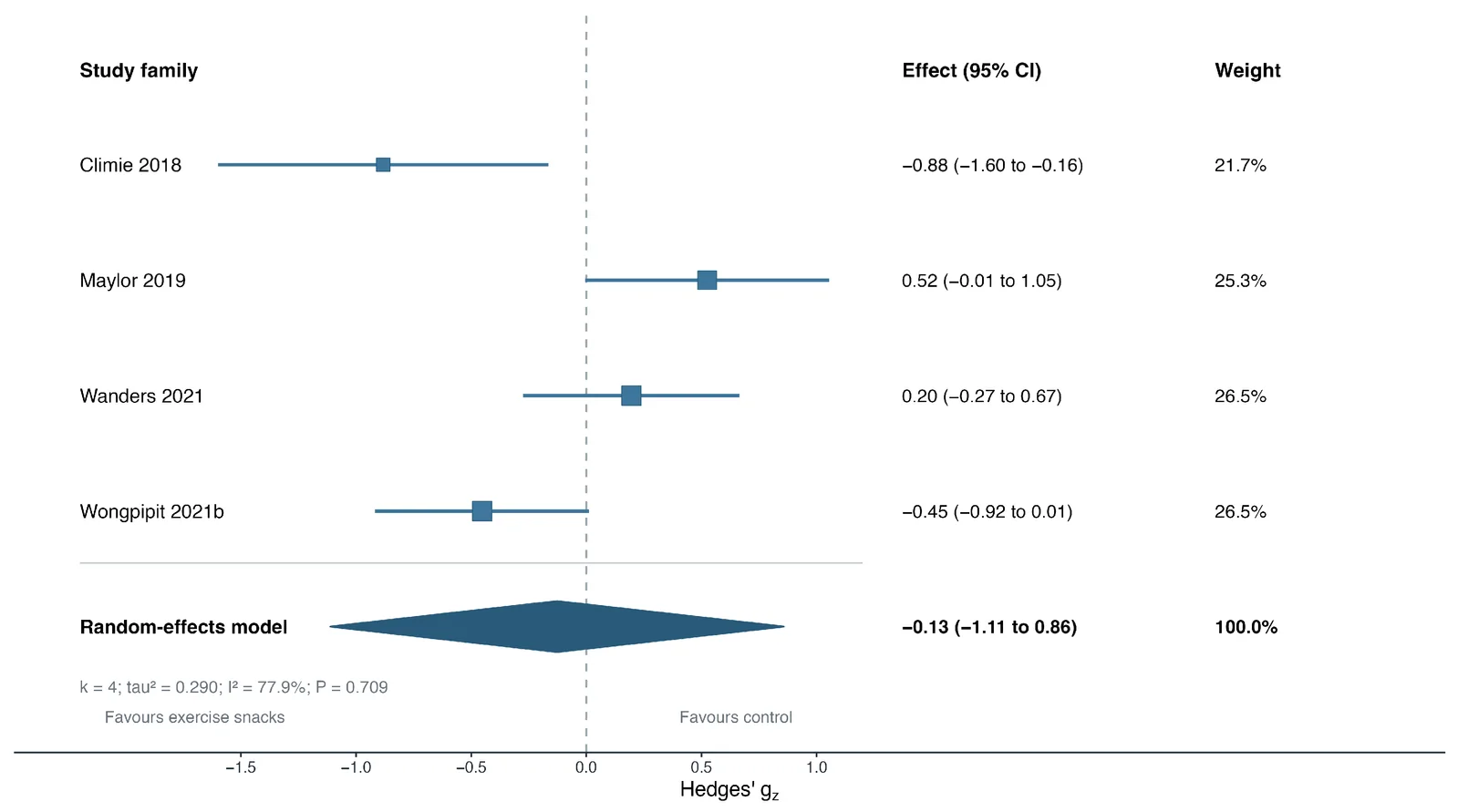
De novo random-effects meta-analysis of acute plasma glucose iAUC. Squares show study-family estimates and are proportional to random-effects weights; horizontal lines indicate 95% confidence intervals, the diamond shows the pooled estimate. The dashed vertical line denotes the null. Random-effects estimates were fitted by REML with Hartung–Knapp adjustment under the primary within-participant correlation assumption (*r* = 0.50). The contributing study families were Wongpipit 2021b [53], Maylor 2019 [54], Climie 2018 [55], and Wanders 2021 [56].

Both the 95% confidence interval and prediction interval included the null, and between-study heterogeneity was substantial. The available evidence therefore did not show a clear pooled effect of exercise snacks on acute plasma glucose iAUC (Figure 3). Given the substantial heterogeneity and wide prediction interval, this finding indicates that a consistent average effect was not established; it does not demonstrate an absence of glucose effects across all intervention settings.

#### 3.3.2. Acute Plasma Insulin iAUC

**Figure 4:**
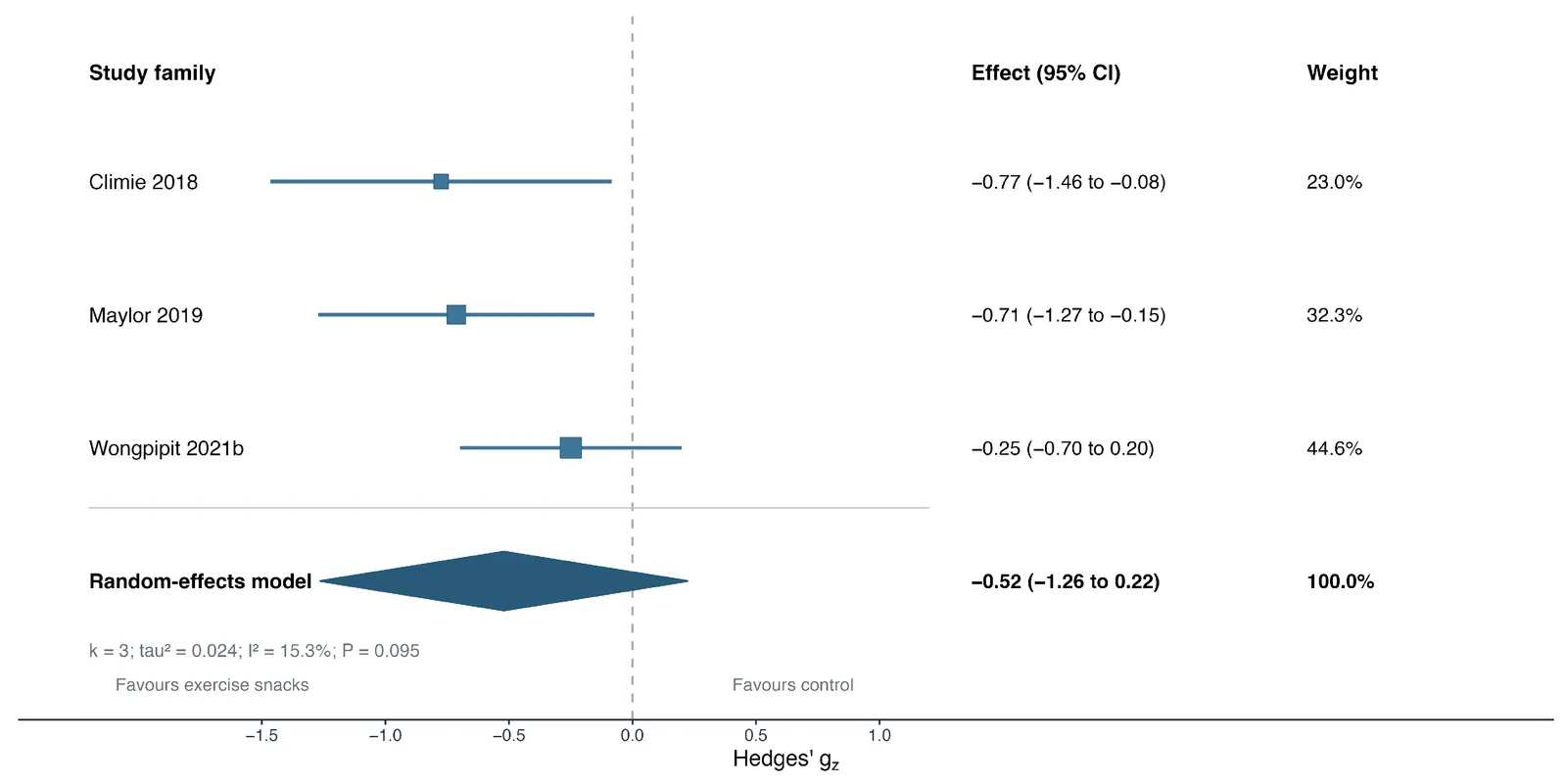
De novo random-effects meta-analysis of acute plasma insulin iAUC. Squares show study-family estimates and are proportional to random-effects weights; horizontal lines indicate 95% confidence intervals, and the diamond shows the pooled estimate. The dashed vertical line denotes the null. Random-effects estimates were fitted by REML with Hartung–Knapp adjustment under the primary within-participant correlation assumption (*r* = 0.50). The contributing study families were Wongpipit 2021b [53], Maylor 2019 [54], and Climie 2018 [55].

Three independent study families contributed to the random-effects meta-analysis of acute plasma insulin iAUC. The pooled Hedges’ *g_z_* was −0.520 (95% CI −1.263 to 0.222; *p* = 0.095), with *τ*^2^ = 0.024, *I*^2^ = 15.3% (*Q*-test *p* = 0.307), and a 95% prediction interval of −1.517 to 0.477. Although the point estimate favored lower insulin iAUC, the confidence interval included the null and was compatible with estimates ranging from a relatively large reduction to an effect close to zero. Thus, no clear pooled effect was observed (Figure 4).

#### 3.3.3. Exploratory and Non-Pooled Metabolic Outcomes

Gale 2023 [24] (*n* = 28) reported plasma glucose and insulin iAUC on a log-ratio scale, with estimates of −0.378 (95% CI −0.644 to −0.113) and −0.304 (95% CI −0.482 to −0.126), respectively. On this scale, the negative estimates and confidence intervals excluding the null indicated lower postprandial glucose and insulin exposure under the exercise-snack condition. Because no second independent study family provided comparable data for the same outcome, measurement medium, and effect scale, these findings were retained as favorable single-study results and were not pooled with the Hedges’ *g_z_* estimates (Supplementary Table S3).

The exploratory acute CGM glucose iAUC analysis included only two study families (*n* = 30). Bailey 2022 [51] showed an estimate close to the null (Hedges’ *g_z_* = −0.025, 95% CI −0.552 to 0.501), whereas Gao 2024 [52] showed a substantially favorable estimate (Hedges’ *g_z_* = −2.143, 95% CI −2.971 to −1.316). The pooled Hedges’ *g_z_* was −1.059 (95% CI −14.510 to 12.392; *p* = 0.500), with *I*^2^ = 94.4%. Given the two-study evidence base, discordant estimates, and extremely wide Hartung–Knapp confidence interval, this analysis was considered descriptive and hypothesis-generating only and neither supported nor refuted an acute CGM glucose effect (Supplementary Figure S1 and Table S2).

#### 3.3.4. Risk of Bias and Certainty of Evidence

Across the three quantitative evidence bodies, 11 study-family–outcome records were assessed with RoB 2. All were rated as some concerns, and none were rated as high risk (Supplementary Table S4 and Figure S4).

GRADE certainty was moderate for acute plasma glucose iAUC, downgraded primarily for serious inconsistency; moderate for acute plasma insulin iAUC, downgraded primarily for serious imprecision; and low for exploratory acute CGM glucose iAUC, downgraded for both serious inconsistency and serious imprecision (Table 2; Supplementary Table S5). Each evidence body contained only 2–4 independent study families, so funnel-plot asymmetry and small-study effects were not formally assessed.

#### 3.3.5. Sensitivity Analyses

Varying the within-participant correlation assumption did not materially change the main interpretation. For plasma glucose iAUC, pooled Hedges’ *g_z_* values were −0.133 (95% CI −1.027 to 0.760) at *r* = 0.25 and −0.105 (95% CI −1.299 to 1.089) at *r* = 0.75. Corresponding estimates for plasma insulin iAUC were −0.456 (95% CI −1.160 to 0.248) and −0.650 (95% CI −1.573 to 0.273) and for exploratory CGM glucose iAUC were −0.867 (95% CI −11.853 to 10.119) and −1.493 (95% CI −20.498 to 17.513). All confidence intervals continued to include the null.

Replacing the WALK arm with the SQUAT arm in Gao 2024 yielded an exploratory CGM estimate of Hedges’ *g_z_* = −0.983 (95% CI −13.437 to 11.471; *I*^2^ = 94.0%), which remained highly imprecise. In leave-one-out analyses, the 95% confidence interval for plasma glucose iAUC included the null regardless of which study family was omitted. The insulin evidence set was more sensitive to study composition: omitting Wongpipit 2021b [53] yielded Hedges’ *g_z_* = −0.737 (95% CI −1.122 to −0.352), whereas omitting either of the other two study families produced confidence intervals that still included the null. This pattern indicates that the insulin estimate was sensitive to study composition and therefore unstable; with only three independent study families, the relatively low *I*^2^ should not be interpreted as evidence of robust consistency.

Fixed-effect sensitivity models produced narrower confidence intervals than the primary Hartung–Knapp random-effects models in some scenarios, and some intervals did not include the null. However, these findings were not consistent across outcomes or sensitivity scenarios and therefore did not replace interpretation of the prespecified random-effects analyses. Complete sensitivity-analysis results are provided in Supplementary Table S8 and Supplementary Figures S2 and S3.

#### 3.3.6. Other Outcome Domains

Blood lipids, blood pressure, physical fitness/physical function, and body composition did not yield sets of at least two independent study families that were sufficiently comparable in population, intervention, outcome definition, measurement method, time window, and effect scale. These domains were therefore not subjected to de novo meta-analysis (Supplementary Table S7). Failure to meet pooling criteria reflects insufficient quantitative comparability, not evidence of no intervention effect.

At the review level, directional signals were reported for blood lipids [11,37,39,48] and blood pressure/vascular outcomes [38,45,57].

Corresponding review-level signals were also reported for physical fitness/physical function [11,47,50] and body composition [39,42,44]. Findings were inconsistent and derived from substantially overlapping primary evidence; overlap was particularly high for the physical-function/older-adult-function review set (CCA = 30.2%). Because these domains were not independently re-synthesized, the reported directions were retained only for evidence mapping and hypothesis generation and should not be interpreted as evidence of efficacy or as multiple independent confirmations of benefit.

## 4. Discussion

The principal finding for postprandial metabolism was a marked discrepancy between the apparent abundance of review-level evidence and the much smaller amount of independent, poolable primary evidence. Although 21 systematic reviews were included and outcome-specific CCA ranged from 11.2% to 30.2%, study-family deduplication and comparability restrictions reduced the main acute metabolic evidence to 4 plasma-glucose, 3 plasma-insulin, and 2 exploratory CGM study families. None of the main random-effects analyses established a statistically clear pooled effect on these acute metabolic responses, and GRADE certainty ranged from moderate to low. However, each synthesis contained only 2–4 independent study families. Under these conditions, the Hartung–Knapp adjustment appropriately accounts for uncertainty in the estimated between-study variance but produces wide confidence intervals and limited statistical power to distinguish small-to-moderate metabolic effects from the null. Importantly, the use of REML estimation, Hartung–Knapp adjustment, prediction intervals, sensitivity analyses, and GRADE improves the transparency and statistical handling of uncertainty but does not increase the number or information content of the underlying independent experiments. The pooled estimates should therefore be viewed primarily as descriptive summaries of a sparse and potentially unstable evidence base rather than as stable estimates of the underlying intervention effect. Accordingly, the absence of a statistically clear pooled effect should not be interpreted as evidence that exercise snacks are metabolically ineffective.

### 4.1. Acute Postprandial Glucose and Insulin Metabolism

For plasma glucose iAUC, both the pooled confidence interval and the prediction interval crossed the null, and between-study heterogeneity was substantial. GRADE certainty was downgraded primarily for serious inconsistency. This does not negate potentially favorable acute responses under specific protocols: randomized crossover trials by Dunstan, Peddie, and Dempsey reported improved postprandial glucose responses in adults with overweight/obesity, healthy adults, and adults with type 2 diabetes, respectively [13,14,15]. However, these studies differed in activity mode, frequency, duration, meal timing, and baseline metabolic status. The pooled result is therefore better interpreted as showing that an average effect across protocols has not been consistently established than as allowing any single positive or non-significant trial to represent the evidence body as a whole. The near-null pooled estimate should therefore not be interpreted as a uniform absence of effect: responses in individual settings may differ in direction and magnitude, although the small evidence base precludes reliable identification of the responsible modifiers.

For plasma insulin iAUC, the pooled point estimate favored lower insulin exposure, but the 95% confidence interval included the null, and the evidence was limited primarily by serious imprecision. The leave-one-out analysis demonstrated pronounced dependence on study composition. Specifically, omitting Wongpipit 2021b [53] reduced heterogeneity from *I*^2^ = 15.3% to 0.0% and shifted the pooled estimate to Hedges’ *g_z_* = −0.737 (95% CI −1.122 to −0.352), whereas omitting either of the other two study families produced confidence intervals that continued to include the null. Thus, both the statistical significance and estimated heterogeneity of the insulin synthesis were structurally dependent on the inclusion of a single study family. Because only two studies remained in the influential leave-one-out model, this result should not be interpreted as confirmatory evidence of benefit; rather, it demonstrates the volatility of inference in a three-study evidence set. We therefore interpret the insulin finding primarily as evidence of continuing uncertainty and study-composition sensitivity rather than as reliable evidence of reduced insulin exposure. The statistically significant estimate obtained after omitting Wongpipit 2021b [53] should not be regarded as confirmatory because only two study families remained, and the relatively low *I*^2^ in the primary analysis does not establish robust consistency in a three-study synthesis.

Gale 2023 [24], the largest single study family in this outcome-specific evidence set (*n* = 28), reported log-ratio estimates for both glucose and insulin iAUC whose confidence intervals excluded the null, indicating lower postprandial exposure under the exercise-snack condition. Its exclusion from the Hedges’ *g_z_* meta-analyses was based solely on incompatibility of the effect scale and the absence of the paired variance information required for a defensible conversion rather than on the direction or statistical significance of its findings. Because log ratios and standardized paired effects are not directly commensurate, the magnitude by which inclusion of Gale 2023 [24] would have shifted the Hedges’ *g_z_* pooled estimates cannot be quantified from the available data. Nevertheless, this favorable result from a comparatively large independent study family adds important directional evidence and further supports interpreting the meta-analytic findings as incomplete and uncertain rather than metabolically neutral.

The acute CGM synthesis was based on only two study families (*n* = 30), with extreme heterogeneity and an extremely wide confidence interval; it should therefore be regarded as descriptive and hypothesis-generating only rather than as evidence supporting or refuting an effect.

The two estimates arose from distinct methodological contexts. Bailey 2022 [51] evaluated a multiday free-living intervention with flexible activity interruptions and a 24 h outcome window. In contrast, Gao 2024 [52] used a controlled 8.5 h laboratory protocol with standardized meals and regularly timed walking bouts. The studies also differed in participant characteristics, activity timing, and the temporal relation between exercise and meals, and both used interstitial CGM measurements, which may temporally lag or smooth plasma glucose changes [58]. These are observed methodological differences, but with only two studies, their contribution to the discordant estimates cannot be determined.

### 4.2. Intervention Definition and Evidence Comparability

Our findings also show that “exercise snacks” cannot yet be treated as a single intervention without regard to dose and organization. Early formulations generally emphasized very brief, relatively high-intensity bouts distributed across the day [3], whereas the recent consensus on short bouts of accumulated exercise further specifies bout duration, daily frequency, and recovery intervals [7]. VILPA focuses on incidental short bursts of vigorous activity in daily life [6], while reviews of prolonged-sitting interruptions have used broader activity modes and frequency structures [8,9,10,36,40,46,49]. These concepts all involve brief, distributed activity, but they differ in physiological stimulus, behavioral context, and prescription intent. To improve comparability in the de novo component, we applied an operational definition requiring active movement, bouts ≤ 15 min, at least two bouts per intervention day, and distribution across the day, while excluding standing-only interruptions and bouts > 15 min. The operational definition therefore excluded some otherwise relevant brief-activity evidence, but it was not the sole explanation for the small quantitative evidence base; study-family overlap and the lack of compatible outcome, measurement-medium, and effect-scale data produced further attrition.

The biochemical stimulus may also differ substantially across eligible exercise-snack protocols. Brief high-intensity bouts can produce a rapid increase in catecholaminergic drive, energetic stress, and contraction-related AMPK signaling, whereas repeated low-to-moderate walking or resistance-based interruptions may generate a smaller acute stress response but more regularly engage skeletal-muscle oxidative metabolism and contraction-mediated, partly insulin-independent GLUT4 translocation [3,13,14,15,16,17]. These pathways are overlapping rather than mutually exclusive, but their relative activation may differ across intervention modes. These observed biochemical differences provide a biologically plausible hypothesis for the substantial inconsistency in plasma glucose iAUC (*I*^2^ = 77.9%); however, with so few independent study families, mode- or intensity-specific effect modification was not tested and cannot be identified as an established driver of heterogeneity.

More broadly, the included studies demonstrably differed in activity mode, intensity, bout duration, daily frequency, inter-bout interval, meal timing, and comparator conditions. These characteristics constitute observed clinical and methodological heterogeneity. By contrast, whether any of them modifies the metabolic effect of exercise snacks remains untested. Although recent reviews have explored potential moderators [40,46,49], overlap in primary studies and the availability of only 2–4 independent study families per quantitative evidence body precluded reliable subgroup analysis or meta-regression. Dose- and protocol-specific relationships should therefore be regarded as hypotheses for future trials rather than established explanations or a basis for prescriptive recommendations. Future studies should distinguish prescribed from achieved dose, systematically report adherence and adverse events, and use CERT and TIDieR to improve intervention reporting [59,60].

### 4.3. Population Differences and Other Health Outcomes

The available evidence includes healthy or physically inactive adults, adults with overweight/obesity or elevated metabolic risk, and older adults, but the evidence base is uneven across populations [11,40,43,50]. In adults with type 2 diabetes, brief activity interruptions have improved postprandial metabolism under controlled experimental conditions, with some effects persisting overnight [15,16]. These observations do not establish disease status itself as a consistent effect modifier. The de novo evidence set remains too small to determine whether health status, adiposity, medication use, habitual physical activity, or body composition systematically modifies responses to exercise snacks. Questions about differential response across populations should therefore remain exploratory rather than being converted into population-specific prescriptions from limited subgroup signals.

Age and sex are also plausible effect modifiers because they may covary with insulin sensitivity, skeletal-muscle mass, adiposity, habitual physical activity, and the magnitude of the postprandial metabolic challenge. However, the present evidence cannot establish age- or sex-specific responses. The contributing study families were few, generally small, and heterogeneous in their age distributions and male–female composition; several recruited narrowly defined or sex-imbalanced samples, including male-only and female-only samples, and sex-stratified effect estimates were generally unavailable (Supplementary Table S11). Age and sex also varied alongside metabolic-risk status, body composition, and intervention protocol, preventing their independent effects from being distinguished. With only 2–4 independent study families per quantitative evidence body, subgroup analysis or meta-regression would have been statistically unreliable and vulnerable to ecological inference. Future trials should recruit adequately balanced samples, report menopausal status where relevant, and provide sex-disaggregated outcomes or interaction estimates.

Evidence for physical fitness/physical function [11,47,50]; blood lipids, blood pressure, and vascular outcomes [38,39,45,48]; and body composition [39,42,44] remained at the review-mapping level because no sufficiently comparable set of at least two independent study families was available for de novo pooling (Supplementary Table S7). Although some favorable directions were reported, the findings were inconsistent and were drawn from overlapping primary studies, including very high overlap for physical function. We therefore do not interpret these review-level signals as evidence of efficacy; they identify domains requiring independent, prospectively harmonized primary studies.

### 4.4. Real-World Implementation and Nutritional–Metabolic Context

The evidence that could be re-pooled was dominated by small acute crossover trials. Such designs are well suited to detecting physiological responses over the hours after a meal, but they cannot be directly extrapolated to long-term HbA1c, cardiovascular events, sustained adherence, or other clinical outcomes. Real-world implementation therefore needs to be considered separately from acute physiological response. Existing trials suggest that brief exercise programs can be feasible and acceptable, but feasibility does not guarantee that the delivered stimulus is sufficient to produce measurable adaptation. In a 12-week technology-enabled randomized trial, Babir et al. [61] showed that physically inactive adults could complete exercise snacks in free-living conditions, yet no clear between-group improvements were observed in VO_2_peak or cardiometabolic biomarkers. Fyfe et al. [62] similarly reported high feasibility and acceptability of remotely delivered home-based resistance exercise snacking in community-dwelling older adults. Conversely, Yin et al. [63] observed improved VO_2_peak after 6 weeks of distributed stair-climbing exercise snacks, and Perkin et al. [64] reported signals of improved lower-limb function in a small older-adult study. Together, these findings suggest that feasibility, adequacy of the training stimulus, and physiological outcomes should be evaluated as distinct dimensions.

Nutritional context was another observed dimension of protocol variation across the included studies. Meal glycemic index and glycemic load can themselves alter postprandial glucose and insulin responses [20], while classic exercise-snack and distributed-walking studies have used different pre- and post-meal activity timing [4,18]. Bellini et al. [19] further showed that meal–exercise timing can influence postprandial glycemia, and Wheeler et al. [65] found that combining continuous exercise with regular activity interruptions can alter postprandial insulin and triglyceride responses, with different metabolic outcomes not necessarily changing in parallel. Meal composition, total energy and carbohydrate exposure, and the interval between eating and activity are therefore relevant design characteristics, but their roles as effect modifiers were not established.

Study-level mapping further demonstrated marked variation in both meal composition and meal–activity timing (Supplementary Table S10). Standardized meals ranged from high-carbohydrate breakfasts and mixed meals of moderate glycemic index to high-energy evening meals, whereas Bailey 2022 [51] used a free-living dietary protocol without a fixed postprandial activity delay. The first eligible activity bout occurred before the first meal in Maylor 2019 [54], approximately 20–30 min after the meal or experimental condition began in Climie 2018 [55] and Wanders 2021 [56], approximately 15–20 min after meal completion in Gale 2023 [24] and Wongpipit 2021b [53], and approximately 45 min after breakfast completion in Gao 2024 [52]. Consequently, the synthesized studies did not expose participants to a uniform nutritional challenge or target the same phase of the postprandial glucose and insulin curves. The observed variation in meal composition and meal–activity timing provides a biologically plausible hypothesis for between-study differences, but it does not establish that either factor modified the intervention effect. Nutrition–exercise snack interactions should therefore be evaluated as prespecified hypotheses in future trials.

## 5. Strengths and Limitations

Several methodological features strengthen this review. First, the umbrella-review framework was combined with de novo analysis at the independent study-family level and an explicit operational definition of exercise snacks, avoiding the assumption that more reviews necessarily represent more independent evidence. Second, six major bibliographic databases and three supplementary literature-discovery sources were systematically searched, while AMSTAR 2, ROBIS, and outcome-specific CCA were used to assess review quality, risk of bias, and evidence overlap. Study-family reconstruction further reduced the risk of double counting primary evidence reported in multiple publications. Third, RoB 2 and GRADE were applied to the de novo evidence; plasma and CGM outcomes were analyzed separately, and fundamentally non-comparable effect scales were not combined. Finally, Hartung–Knapp random-effects models, prediction intervals, and multiple sensitivity analyses were used to make modeling uncertainty explicit and to limit reliance on any single statistical assumption. These procedures strengthen the analytical handling of sparse evidence but cannot compensate for the very small number of independent study families or make the resulting effect estimates intrinsically stable.

The study also has important limitations. First, primary-study overlap across review sets was high, so review counts overstate the amount of independent evidence. Second, the de novo evidence base was small and consisted mainly of acute crossover trials; all 11 study-family–outcome RoB 2 records were rated as some concerns. Third, differences in intervention definitions, doses, measurement media, effect scales, comparator conditions, and outcome time points substantially reduced the number of studies that could be pooled reliably. Fourth, too few independent study families were available to meaningfully assess funnel-plot asymmetry or small-study effects, and publication bias therefore cannot be excluded. Fifth, 14 of 73 reports sought for retrieval were not obtained, which may have reduced the completeness of accessible evidence. Finally, the main quantitative outcomes were acute physiological measures, limiting inference about long-term clinical outcomes and real-world generalizability; available feasibility and short-term training studies also remain too heterogeneous in population and prescription to support broad extrapolation. In addition, the search strategy was primarily review focused. Although reference-list checking, cross-review reconciliation, and the final search update were used to identify potentially omitted primary reports, an eligible trial not captured by or cited in the included reviews may have been missed.

## 6. Conclusions

The exercise-snack literature contains many systematic reviews but substantial overlap in the underlying primary evidence. For acute postprandial metabolism, study-family deduplication and restriction to quantitatively comparable evidence showed no clear pooled effect on plasma glucose or insulin iAUC, while the two-study exploratory CGM synthesis was considered descriptive and hypothesis-generating only and did not inform conclusions regarding efficacy. The evidence therefore supports a conclusion of limited independent evidence and persistent uncertainty regarding these metabolic responses rather than a definitive claim that exercise snacks are metabolically ineffective. Accordingly, the pooled estimates should be interpreted as descriptive summaries of the currently available independent evidence rather than as precise or stable estimates of average intervention effects. More robust inference will require a larger, more comparable, and more independent primary evidence base with standardized characterization of postprandial metabolic outcomes. The insulin estimate was particularly sensitive to study composition and should not be interpreted as stable evidence of reduced postprandial insulin exposure. For glucose, this indicates failure to establish a consistent average effect across heterogeneous protocols rather than evidence of a uniform absence of effect.

## Figures and Tables

**Table 1:** The characteristics and quality appraisal of the 21 included systematic reviews.

Author (Year)	Review Focus	Population	Evidence Base	Outcome Domains	AMSTAR 2	ROBIS
Shambrook et al. (2018) [34]	Accumulated vs. continuous aerobic exercise	Inactive adults, 19–64 y; no diagnosed glucose disorder	149 reports; 16 RCTs in meta-analysis	Glycemia	Moderate	Unclear
Murphy et al. (2019) [35]	Accumulated vs. continuous exercise (dose-matched)	Community-dwelling adults	19 studies; *n* = 1080	Fitness; BP; lipids; glycemia; body composition	High	Low
Loh et al. (2020) [8]	Sitting interruptions: active breaks	Adults with normal or impaired metabolic health	42 studies; 37 in meta-analysis	Glucose; insulin; TG	Moderate	Low
Quan et al. (2021) [36]	Sitting interruptions: network meta-analysis	Adults without chronic disease	30 crossover RCTs	Postprandial glucose; insulin	Moderate	Low
Buffey et al. (2022) [9]	Sitting interruptions: standing/light walking	Adults	7 studies	Glucose; insulin; SBP; other cardiometabolic markers	Moderate	Unclear
Zhang et al. (2022) [37]	Accumulated vs. continuous exercise (energy-matched)	Adults with or without diabetes	27 studies; *n* = 635	Postprandial glucose; insulin; TG	High	Low
Paterson et al. (2022) [38]	Acute sitting interruptions	Adults aged ≥ 18 y	33 studies; 22 sitting-break studies	SBP; DBP; MAP	Moderate	Unclear
Chang et al. (2025) [43]	Acute exercise snacks/activity breaks	Adults with obesity	17 trials; *n* = 261	Postprandial glucose; insulin	High	Low
Wan et al. (2025) [39]	Exercise snacks: cardiometabolic health/body composition	Adults	14 studies; *n* = 483; 13 in meta-analysis	VO_2_max; peak power; lipids; body composition	Moderate	Unclear
Yin et al. (2025) [40]	Sitting interruptions: multilevel meta-analysis	Middle-aged/older adults (typically ≥45 y)	27 studies	Glucose; insulin; lipids	Moderate	Unclear
Alexe et al. (2025) [41]	Exercise snacks: health outcomes/feasibility	Healthy, older, obesity, T2D and PCOS populations	26 studies	Metabolic; BP/vascular; fitness/function; cognition; feasibility	Low	High
Chen et al. (2025) [42]	Exercise snacks: cardiometabolic health	Adults	27 studies; *n* = 970	VO_2_max; body composition; BP; fasting glucose; lipids	High	Unclear
Lin et al. (2026) [44]	Exercise snacks: body composition	Adults	8 reports/9 studies; *n* = 292	Lean mass; body fat; body mass	Moderate	Unclear
Wang et al. (2026) [45]	Exercise snacks/activity breaks: three-level meta-analysis	Adults aged ≥ 18 y	36 studies; *n* = 632	FMD; blood flow; shear rate; BP	Moderate	Low
Zhuang et al. (2026) [46]	Acute sitting interruptions: three-level meta-analysis	Adults without established cardiometabolic disease	25 reports/23 independent cohorts	Glucose; insulin; TG; NEFA; C-peptide	Moderate	Low
Rodríguez et al. (2026) [11]	Exercise snacks: fitness/cardiometabolic health	Physically inactive adults and older adults	11 RCTs; *n* = 414	Cardiorespiratory fitness; muscular endurance/strength; BP; lipids; body composition	Moderate	Low
Zhang et al. (2026) [47]	Exercise snacks: physical function	Healthy or subhealthy adults	11 RCTs; *n* = 472	Peak power; VO_2_max; 60-s sit-to-stand; body fat; fatigue; BMI	Moderate	Unclear
Peng et al. (2026) [48]	Low-/moderate-intensity exercise snacks	Sedentary adults	15 studies; *n* = 334	Fasting glucose/insulin; TC; TG; LDL-C; HDL-C	Moderate	Low
Vanherle et al. (2026) [49]	Sitting interruptions: different activity modes	Adults 18–65 y; with or without cardiometabolic conditions	144 studies/247 intervention arms; *n* = 2216	Glucose; TG; endothelial function; related outcomes	High	Low
Gale et al. (2026) [10]	Brief activity interruptions to sitting	Adults	53 studies; 39 in meta-analysis	Postprandial glucose; insulin	Low	Low
Zhang et al. (2026) [50]	Remote exercise snacks: fall-related function	Physically inactive older adults (~65–75 y)	4 reports/10 studies; *n* = 313	Lower-limb strength/endurance; balance; adherence/acceptability	Moderate	Unclear

Note. Review focus and outcome domains were harmonized for concise presentation; eligibility definitions and study counts remain those reported in the original systematic reviews. A single review may contribute to multiple outcome domains. AMSTAR 2 and ROBIS ratings are the quality-appraisal results of the present umbrella review. BP, blood pressure; DBP, diastolic blood pressure; FMD, flow-mediated dilation; HDL-C, high-density lipoprotein cholesterol; LDL-C, low-density lipoprotein cholesterol; MAP, mean arterial pressure; NEFA, non-esterified fatty acids; PCOS, polycystic ovary syndrome; RCT, randomized controlled trial; SBP, systolic blood pressure; T2D, type 2 diabetes; TC, total cholesterol; TG, triglycerides; VO_2_max, maximal oxygen uptake.

**Table 2:** GRADE summary of findings for the de novo meta-analyses.

Outcome	*k*	*n*	Pooled Hedges’ *g_z_*	95% CI	*I* ^2^	GRADE Certainty	Interpretation
Acute plasma glucose iAUC	4	57	−0.127	−1.111 to 0.857	77.9%	Moderate	No clear pooled effect; the interval was compatible with benefit and effects close to the null.
Acute plasma insulin iAUC	3	41	−0.520	−1.263 to 0.222	15.3%	Moderate	No clear pooled effect; the confidence interval included the null and the estimate remained imprecise.
Exploratory acute CGM glucose iAUC	2	30	−1.059	−14.510 to 12.392	94.4%	Low	Direction and magnitude were highly uncertain; exploratory interpretation only.

Note. Random-effects models used REML with Hartung–Knapp adjustment. *k*, number of independent study families; *n*, total participants; CI, confidence interval; iAUC, incremental area under the curve; CGM, continuous glucose monitoring; GRADE, Grading of Recommendations Assessment, Development and Evaluation.

## Data Availability

The original contributions presented in this study are included in the article and Supplementary Materials. Further inquiries can be directed to the corresponding author.

## Supplementary Materials

The following supporting information can be downloaded at https://www.mdpi.com/article/10.3390/metabo16090635/s1, Figure S1: De novo random-effects meta-analysis of exploratory acute CGM glucose iAUC; Figure S2: Random-effects sensitivity analyses for within-participant correlation assumptions and the alternative Gao 2024 intervention arm; Figure S3: Replacement-arm and leave-one-out sensitivity analyses for the de novo metabolic syntheses; Figure S4: Traffic-light plot of RoB 2 judgments for study-family–outcome records contributing to the de novo quantitative evidence; Table S1: Operational definition and eligibility framework for the de novo component; Table S2: Study-family estimates entering the primary paired Hedges’ *g*_z_ de novo analyses; Table S3: Single-study log-ratio estimates retained but not pooled; Table S4: Detailed RoB 2 judgments by study family and outcome; Table S5: Detailed GRADE evidence profile; Table S6: Outcome-specific corrected covered area (CCA) for review-level evidence overlap; Table S7: Outcome domains that did not meet criteria for de novo quantitative synthesis; Table S8: Complete sensitivity-analysis results; Table S9: Post-registration methodological refinements and transparency status; Table S10: Test-meal composition and meal–activity timing in primary study families contributing to the acute metabolic synthesis; Table S11: Sex composition of primary study families contributing to the acute metabolic synthesis; File S1: Full electronic search strategies.

## Abbreviations

The following abbreviations are used in this manuscript:

AMSTAR 2	A Measurement Tool to Assess Systematic Reviews 2
BP	Blood pressure
CCA	Corrected covered area
CERT	Consensus on Exercise Reporting Template
CGM	Continuous glucose monitoring
CI	Confidence interval
DBP	Diastolic blood pressure
FMD	Flow-mediated dilation
GRADE	Grading of Recommendations Assessment, Development and Evaluation
HDL-C	High-density lipoprotein cholesterol
iAUC	Incremental area under the curve
LDL-C	Low-density lipoprotein cholesterol
MAP	Mean arterial pressure
NEFA	Non-esterified fatty acids
PCOS	Polycystic ovary syndrome
PICOS	Population, Intervention, Comparator, Outcomes, Study design
PRIOR	Preferred Reporting Items for Overviews of Reviews
PRISMA	Preferred Reporting Items for Systematic Reviews and Meta-Analyses
PROSPERO	International Prospective Register of Systematic Reviews
RCT	Randomized controlled trial
REML	Restricted maximum likelihood
RoB 2	Risk of Bias 2
ROBIS	Risk of Bias in Systematic Reviews
SBP	Systolic blood pressure
T2D	Type 2 diabetes
TC	Total cholesterol
TG	Triglycerides
TIDieR	Template for Intervention Description and Replication
VILPA	Vigorous intermittent lifestyle physical activity
VO_2_max	Maximal oxygen uptake
