# Supplementary material for: Exercise Snacks and Acute Postprandial Glucose and Insulin Metabolism: An Umbrella Review and De Novo Meta-Analysis
Source: Metabolites. 2026 Aug 31;16(9):635. doi: 10.3390/metabo16090635 (PMC13609052; doi:10.3390/metabo16090635)
Supplement: Supplementary file 1 [file metabolites-16-00635-s001.zip › metabolites-4529682-supplementary.pdf]

Supplementary Material

This supplementary file accompanies the umbrella review and de novo study-family meta-analysis of exercise snacks and related active interruptions of prolonged sitting. All effect estimates and meta-analytic values are drawn from the locked analysis dataset finalized on 7 August 2026. Additional descriptive study-level characteristics extracted during revision are reported in Supplementary Tables S10–S11 and did not alter the locked quantitative analyses. The PROSPERO record is CRD420261460040; the post-registration methodological refinements described in the manuscript have been synchronized with the PROSPERO record and are summarized transparently in Supplementary Table S9.

Supplementary Methods

Operational definition and eligibility framework

For the de novo component, an eligible exercise-snack exposure required intentional active physical activity, a bout duration of 15 min or less, at least two bouts per intervention day, and distribution of bouts across the day. Standing-only interruptions, a single bout per day, and bouts longer than 15 min were excluded. Active interruptions of prolonged sitting were eligible only when they satisfied the same active-movement, duration, frequency, and within-day distribution criteria. Full operational criteria are summarized in Supplementary Table S1.

Search strategy

Six bibliographic databases—PubMed/MEDLINE, Embase, Web of Science Core Collection, Scopus, the Cochrane Database of Systematic Reviews, and SPORTDiscus—and three supplementary literature-discovery sources (Europe PMC, OpenAlex, and Crossref) were searched through 5 August 2026. Search terms were intentionally broader than the final operational definition so that earlier terminology such as accumulated exercise, brief exercise bouts, activity breaks, and breaking up prolonged sitting would not be missed. Eligibility restrictions were applied during screening rather than by outcome, population, or age terms in the search. The complete source-specific search strategies and query syntax are reproduced in Supplementary File S1.

Study-family identification and quantitative hierarchy

Original reports were mapped to independent study families using DOI, author-year, trial or registration identifiers when available, participant and setting characteristics, intervention details, and other cohort-level information. The statistical unit for de novo synthesis was the independent study family rather than the publication. Multiple reports from the same underlying experiment were therefore not counted as independent evidence. Study-family estimates entering the primary paired Hedges' g_z pools are listed in Supplementary Table S2; results reported on an incompatible log-ratio scale and retained as single-study evidence are listed in Supplementary Table S3.

Effect-size derivation and meta-analysis

Paired continuous outcomes from crossover trials were expressed as Hedges' g_z. When the within-participant variance was unavailable, the primary calculation assumed a within-participant correlation of $r = 0.50$; sensitivity analyses used $r = 0.25$ and $r = 0.75$. For Climie 2018, the paired standard deviation was reconstructed from the reported P value before calculating Hedges' g_z. Random-effects models used restricted maximum likelihood estimation with Hartung–Knapp confidence intervals when estimable. Fixed-effect models were retained only as sensitivity analyses and did not replace the prespecified random-effects interpretation. Prediction intervals were reported when informative and estimable.

Multi-arm and robustness rules

Shared comparators were not counted twice. For Gao 2024, the WALK arm was used in the primary exploratory CGM analysis and the SQUAT arm was substituted in a prespecified replacement-arm sensitivity analysis; the two intervention arms were never entered simultaneously with the same comparator. Robustness analyses examined $r = 0.25$, 0.50, and 0.75, the Gao replacement arm, leave-one-out analyses for the plasma pools, and fixed-effect models. Figure S2 summarizes the within-participant correlation and replacement-arm sensitivity estimates across outcomes, whereas Figure S3 presents the Gao 2024 replacement-arm comparison alongside the leave-one-out analyses for the plasma glucose and insulin pools, and Supplementary Table S8 reports the complete numerical results.

Risk of bias, certainty of evidence, and overlap

RoB 2 was applied at the study-family–outcome level to the evidence entering de novo quantitative synthesis. Eleven locked study-family–outcome records were assessed, all with an overall judgment of Some concerns and none with High risk. Detailed domain-level judgments are provided in Supplementary Table S4 and visualized in Supplementary Figure S4. GRADE certainty was assessed separately for acute plasma glucose iAUC, acute plasma insulin iAUC, and exploratory acute CGM glucose iAUC; detailed domain judgments are shown in Supplementary Table S5. Review-level overlap was quantified using outcome-specific corrected covered area (CCA), summarized in Supplementary Table S6.

Supplementary Figures

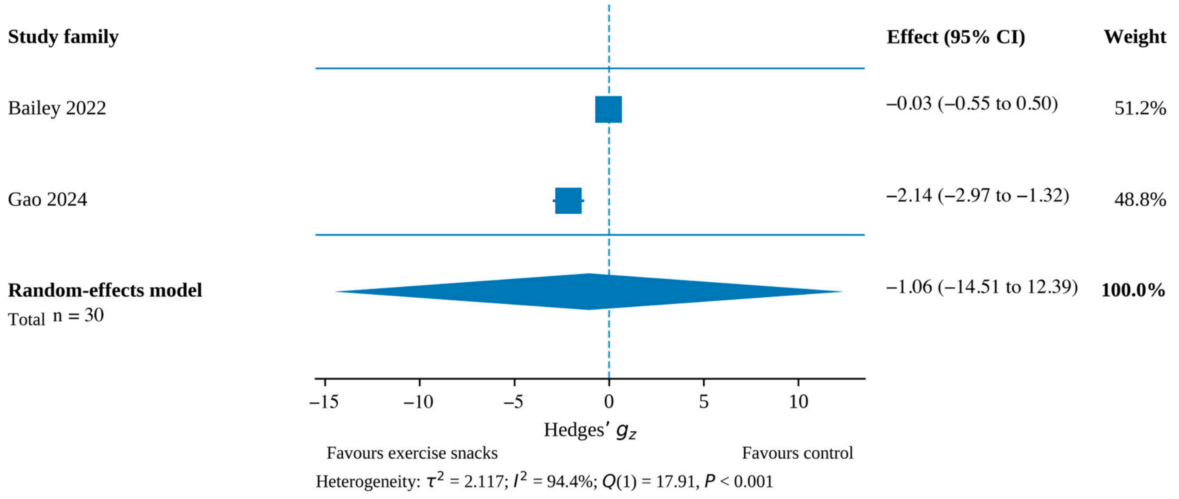

Supplementary Figure S1. De novo random-effects meta-analysis of exploratory acute CGM glucose iAUC.

Squares show study-family estimates and are proportional to random-effects weights; horizontal lines indicate 95% confidence intervals; the diamond shows the pooled estimate. The dashed vertical line denotes the null. The analysis used REML with Hartung–Knapp adjustment under the primary within-participant correlation assumption (r = 0.50). Negative Hedges' g_z values indicate lower CGM glucose iAUC under the exercise-snack/active-interruption condition than under the comparator. Because only two independent study families were available and heterogeneity was very high, this analysis is exploratory.

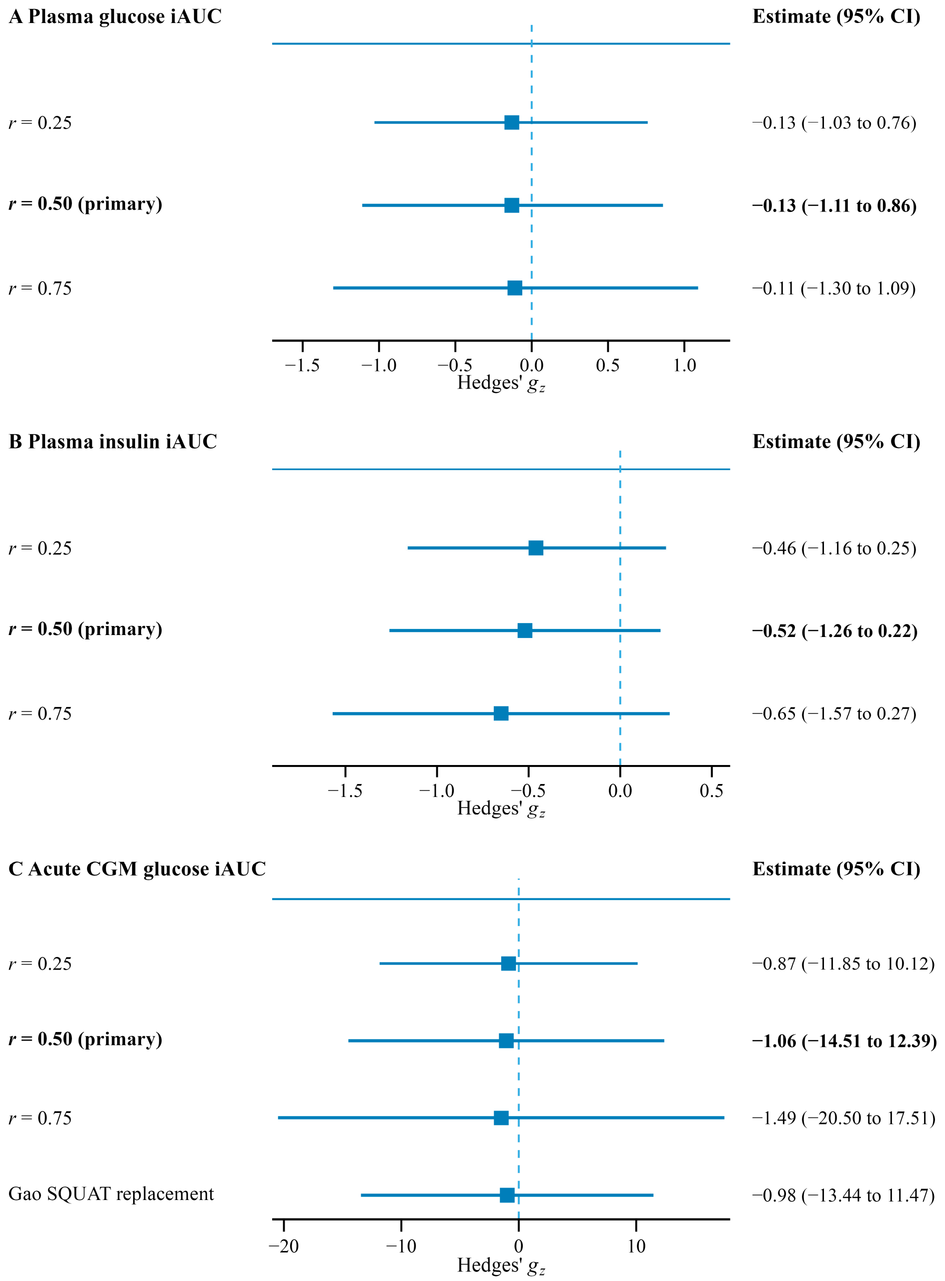

Supplementary Figure S2. Random-effects sensitivity analyses for within-participant correlation assumptions and the alternative Gao 2024 intervention arm.

Panels show Hartung–Knapp random-effects estimates for plasma glucose iAUC, plasma insulin iAUC, and exploratory acute CGM glucose iAUC under $r = 0.25$, 0.50, and 0.75. For CGM, the Gao 2024 SQUAT arm is additionally shown as a replacement for the primary WALK arm. The primary $r = 0.50$ rows are bold. Confidence intervals crossing zero indicate that no clear pooled effect was established under those scenarios.

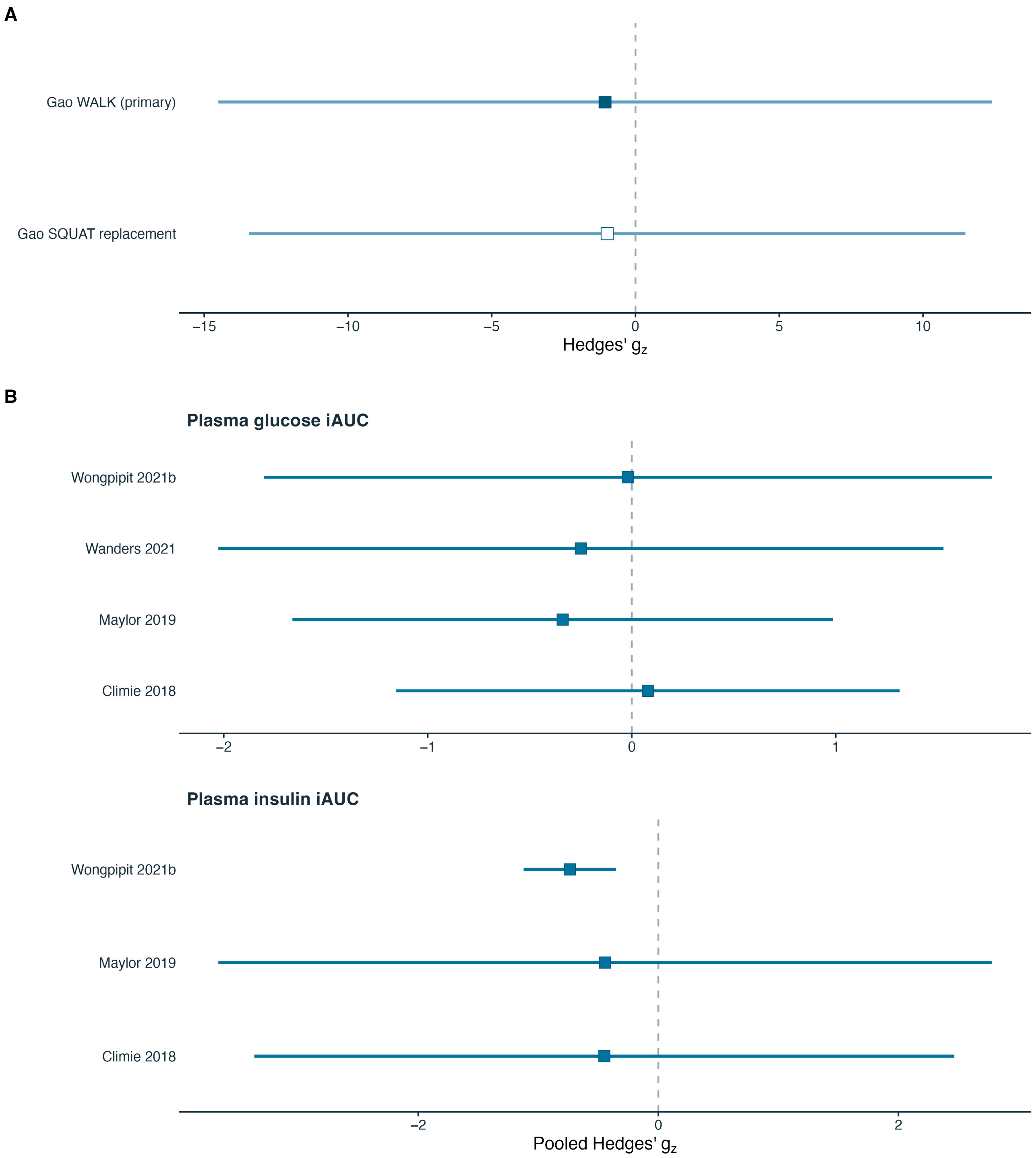

Supplementary Figure S3. Replacement-arm and leave-one-out sensitivity analyses for the de novo metabolic syntheses.

(A) Comparison of the exploratory acute CGM glucose iAUC random-effects estimates using the primary Gao 2024 WALK arm and the alternative SQUAT replacement arm. The two intervention arms were evaluated separately and were never entered simultaneously with the shared comparator.
(B) Leave-one-out random-effects analyses for plasma glucose and plasma insulin iAUC. Each row shows the Hartung–Knapp random-effects estimate after omitting one study family. All plasma glucose leave-one-out confidence intervals included the null. For plasma insulin, omission of Wongpipit 2021b yielded Hedges' $gz = −0.737$ (95% CI −1.122 to −0.352). Because only two

study families remained in this sensitivity analysis, this result indicates sensitivity of the small evidence set to study composition rather than confirmatory evidence of efficacy. The dashed vertical line denotes the null.

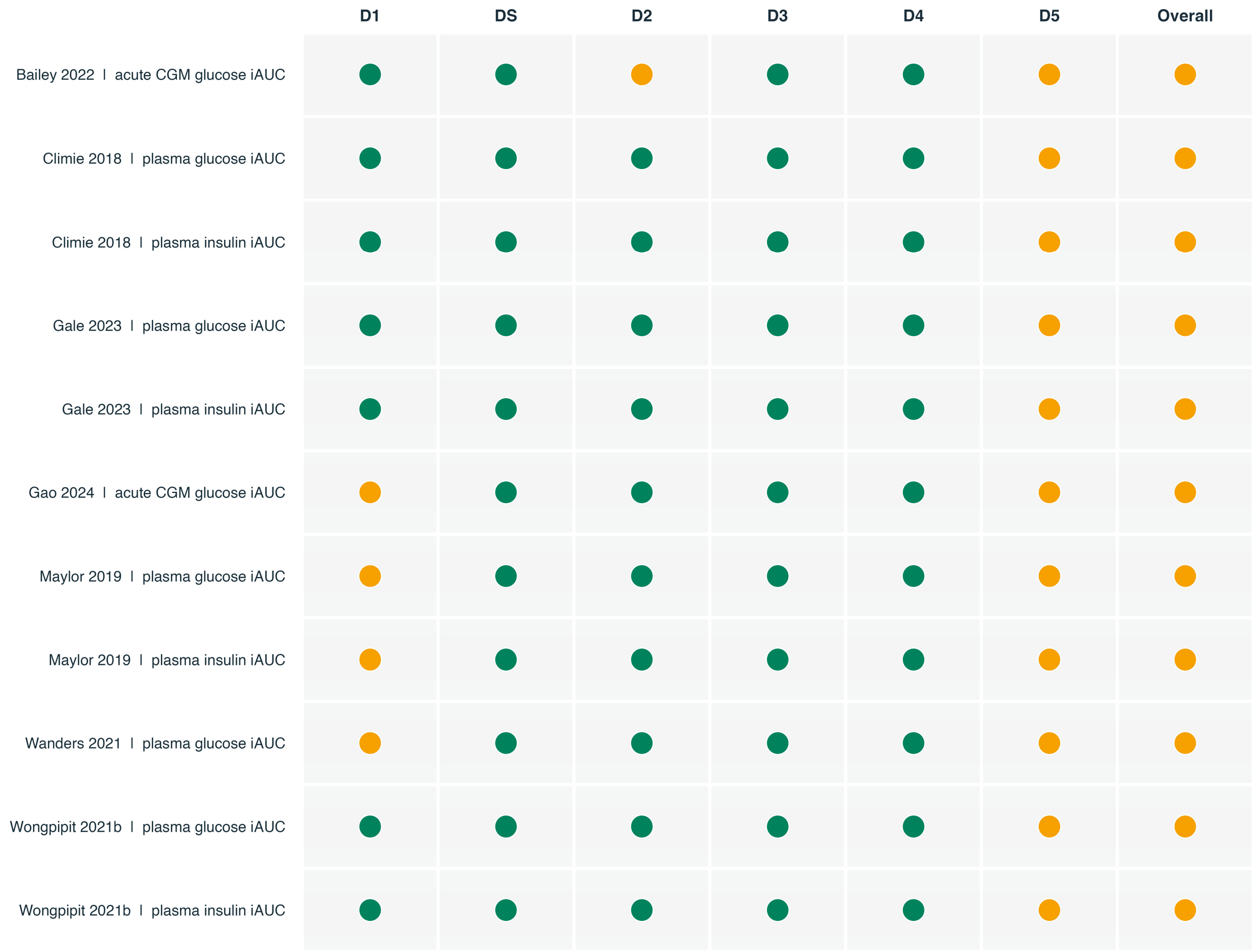

Supplementary Figure S4. Traffic-light plot of RoB 2 judgments for study-family–outcome records contributing to the de novo quantitative evidence.

Risk of bias was assessed at the study-family–outcome level. D1, bias arising from the randomization process; DS, bias arising from period/carryover effects in the crossover design; D2, bias due to deviations from intended interventions; D3, bias due to missing outcome data; D4, bias in measurement of the outcome; D5, bias in selection of the reported result. Green indicates low risk of bias, yellow indicates some concerns, and red indicates high risk of bias. All 11 study-family–outcome records were rated as Some concerns overall; none was rated as high risk.

Supplementary Tables

Supplementary Table S1. Operational definition and eligibility framework for the de novo component

Domain	Criterion
Population	Adults aged ≥18 years; healthy, physically inactive, overweight/obese, metabolically at risk, chronic-disease, older, frail, or musculoskeletal populations were eligible when the intervention/outcome criteria were met.
Core intervention definition	Intentional active physical activity; each bout ≤15 min; ≥2 bouts per intervention day; bouts distributed across the day.
Bout separation	Successive bouts were required to be clearly discrete and distributed across the day rather than forming one continuous or clustered exercise session; no fixed minimum inter-bout interval was imposed.
Eligible modes	Walking, stair climbing, resistance-type movements, sit-to-stand, cycling, mixed exercise, or other purposeful active movement.
Active sitting interruptions	Eligible only when they met the same active-movement, bout-duration, daily-frequency, and within-day distribution criteria.
Key exclusions	Standing-only interruptions; one bout/day; any bout >15 min; traditional continuous exercise; conventional HIIT/SIT delivered as one training session; passive wearable-observed activity without a prescribed intervention.
Comparator	Uninterrupted sitting, usual activity/no intervention, continuous exercise, alternative exercise-snack doses or modes, or other active comparators.
Outcome scope	Cardiometabolic outcomes, physical fitness/function, body composition, and implementation/safety outcomes at review level; de novo pooling required outcome-specific quantitative comparability.
De novo pooling threshold	At least two independent study families sufficiently comparable in population, intervention, comparator, outcome definition, measurement medium, time window, and effect scale.

Note. The search strategy was intentionally broader than these final eligibility rules; strict bout duration, frequency, active-movement, and within-day distribution criteria were applied during screening and original-study re-evaluation.

Supplementary Table S2. Study-family estimates entering the primary paired Hedges' g_z de novo analyses

Outcome	Study family	Medium	*n*	Hedges' g_z	SE	95% CI	Variance source
Acute CGM glucose iAUC	Bailey 2022	CGM	12	-0.025	0.269	-0.552 to 0.501	Assumed r = 0.50
Acute CGM glucose iAUC	Gao 2024	CGM	18	-2.143	0.422	-2.971 to -1.316	Assumed r = 0.50
Plasma glucose iAUC	Climie 2018	plasma	9	-0.882	0.366	-1.599 to -0.165	Derived from reported *P* value
Plasma glucose iAUC	Maylor 2019	plasma	14	0.525	0.270	-0.005 to 1.055	Assumed r = 0.50
Plasma glucose iAUC	Wanders 2021	plasma	16	0.196	0.240	-0.274 to 0.666	Assumed r = 0.50
Plasma glucose iAUC	Wongpipit 2021b	plasma	18	-0.453	0.237	-0.918 to 0.013	Assumed r = 0.50
Plasma insulin iAUC	Climie 2018	plasma	9	-0.775	0.352	-1.465 to -0.084	Derived from reported *P* value
Plasma insulin iAUC	Maylor 2019	plasma	14	-0.713	0.285	-1.272 to -0.153	Assumed r = 0.50
Plasma insulin iAUC	Wongpipit 2021b	plasma	18	-0.249	0.229	-0.698 to 0.199	Assumed r = 0.50

Note. Negative Hedges' g_z values indicate a lower outcome under the exercise-snack/active-interruption condition. For Gao 2024, the WALK arm was used in the primary CGM analysis; SQUAT was used only in a replacement-arm sensitivity analysis. Values shown here are the primary r = 0.50 study-level estimates.

Supplementary Table S3. Single-study log-ratio estimates retained but not pooled

Outcome	Study family	Effect scale	*n*	Estimate	95% CI	Reason not pooled
Plasma glucose iAUC	Gale 2023	log_ratio	28	-0.378	-0.644 to -0.113	Only one independent study in this outcome/medium/effect-scale pool
Plasma insulin iAUC	Gale 2023	log_ratio	28	-0.304	-0.482 to -0.126	Only one independent study in this outcome/medium/effect-scale pool

Note. These estimates were not combined with paired Hedges' g_z because the effect scales are not directly comparable. They remain single-study findings and do not replace the corresponding random-effects meta-analyses.

Supplementary Table S4. Detailed RoB 2 judgments by study family and outcome

Study family	Outcome	D1	DS	D2	D3	D4	D5	Overall
Bailey 2022	acute CGM glucose iAUC	Low	Low	Some concerns	Low	Low	Some concerns	Some concerns
Climie 2018	plasma glucose iAUC	Low	Low	Low	Low	Low	Some concerns	Some concerns
Climie 2018	plasma insulin iAUC	Low	Low	Low	Low	Low	Some concerns	Some concerns
Gale 2023	plasma glucose iAUC	Low	Low	Low	Low	Low	Some concerns	Some concerns
Gale 2023	plasma insulin iAUC	Low	Low	Low	Low	Low	Some concerns	Some concerns
Gao 2024	acute CGM glucose iAUC	Some concerns	Low	Low	Low	Low	Some concerns	Some concerns
Maylor 2019	plasma glucose iAUC	Some concerns	Low	Low	Low	Low	Some concerns	Some concerns
Maylor 2019	plasma insulin iAUC	Some concerns	Low	Low	Low	Low	Some concerns	Some concerns
Wanders 2021	plasma glucose iAUC	Some concerns	Low	Low	Low	Low	Some concerns	Some concerns
Wongpipit 2021b	plasma glucose iAUC	Low	Low	Low	Low	Low	Some concerns	Some concerns
Wongpipit 2021b	plasma insulin iAUC	Low	Low	Low	Low	Low	Some concerns	Some concerns

Note. D1, bias arising from the randomization process; DS, bias arising from period/carryover effects in the crossover design; D2, bias due to deviations from intended interventions; D3, bias due to missing outcome data; D4, bias in measurement of the outcome; D5, bias in selection of the reported result. All 11 locked study-family–outcome records had an overall judgment of Some concerns; none was High risk. CQ and SY both completed the locked assessment.

Supplementary Table S5. Detailed GRADE evidence profile

Evidence body	*k*	*n*	Hedges' g_z	95% CI	95% prediction interval	Certainty
Plasma glucose iAUC	4	57	-0.127	-1.111 to 0.857	-2.103 to 1.849	Moderate
Plasma insulin iAUC	3	41	-0.520	-1.263 to 0.222	-1.517 to 0.477	Moderate
Acute CGM glucose iAUC	2	30	-1.059	-14.510 to 12.392	Not estimable	Low

Note. Randomized crossover evidence was initially rated High and downgraded according to the locked GRADE judgments shown below.

Evidence body	Risk of bias	Inconsistency	Indirectness	Imprecision	Publication bias	Other considerations
Plasma glucose iAUC	Not serious: all four included result-level evaluations had Some concerns; none was high risk	Serious: $I2$=77.9%; estimates differ in direction and magnitude	Not serious: direct acute postprandial plasma outcome after eligible brief active interruptions versus sitting	Not additionally downgraded: wide CI acknowledged; no validated MID prespecified and sparse heterogeneous evidence already drove inconsistency downgrade	Not downgraded: *k*=4 insufficient for reliable asymmetry testing	No upgrade; Wongpipit 2021a excluded because full text unavailable

Evidence body	Risk of bias	Inconsistency	Indirectness	Imprecision	Publication bias	Other considerations
Plasma insulin iAUC	Not serious: all three included result-level evaluations had Some concerns; none was high risk	Not serious: $I2$=15.3%; estimates broadly compatible but interpreted cautiously because k=3	Not serious: direct acute postprandial plasma outcome after eligible brief active interruptions versus sitting	Serious: CI includes appreciable benefit and little or no effect	Not downgraded: k=3 insufficient for reliable asymmetry testing	No upgrade; Wongpipit 2021a excluded because full text unavailable
Acute CGM glucose iAUC	Not serious: both included result-level evaluations had Some concerns rather than high risk	Serious: two markedly discordant estimates; $I2$=94.4%, interpreted cautiously at k=2	Not serious: direct acute interstitial glucose iAUC after eligible exercise-snack patterns	Serious: Hartung–Knapp CI extremely wide; no additional double counting of the same two-study discordance	Not downgraded: k=2 insufficient for reliable asymmetry testing	No upgrade; unaffected by full-text exclusion rule

Note. Publication bias was not downgraded because k = 2–4 was insufficient for meaningful asymmetry testing; this must not be interpreted as evidence that publication bias was absent.

Supplementary Table S6. Outcome-specific corrected covered area (CCA) for review-level evidence overlap

Outcome cluster	Reviews	CCA	Overlap classification	Audit note
Physical function / older-adult function	4	30.2%	Very high	Outcome-specific primary-evidence source matrix
Physical fitness and cardiometabolic health	5	14.4%	High	Outcome-specific primary-evidence source matrix
Glucose / insulin outcomes	6	13.4%	High	73 unique study families / 122 study occurrences
Body composition	3	11.2%	High	Outcome-specific primary-evidence source matrix

Note. CCA quantifies duplicate primary evidence across reviews and is not an intervention effect measure. For glucose/insulin outcomes, the locked audit contained 73 unique study families appearing 122 times across six review-level evidence sources.

Supplementary Table S7. Outcome domains that did not meet criteria for de novo quantitative synthesis

Outcome domain	De novo meta-analysis	Reason
Blood lipids	No	No eligible quantitative effect set contained ≥2 independent study families sufficiently comparable for reliable re-pooling in the locked dataset.
Blood pressure	No	No eligible quantitative effect set contained ≥2 independent study families sufficiently comparable for reliable re-pooling in the locked dataset.
Physical fitness	No	No eligible quantitative effect set contained ≥2 independent study families sufficiently comparable for reliable re-pooling in the locked dataset.
Physical function	No	No eligible quantitative effect set contained ≥2 independent study families sufficiently comparable for reliable re-pooling in the locked dataset.
Body composition	No	No eligible quantitative effect set contained ≥2 independent study families sufficiently comparable for reliable re-pooling in the locked dataset.

Note. Failure to meet de novo pooling criteria indicates insufficient quantitative comparability and must not be interpreted as evidence of no intervention effect.

Supplementary Table S8. Complete sensitivity-analysis results

Outcome	Sensitivity analysis	Omitted family	Model	k	Hedges' g_z	95% CI	P value	τ^2	I^2
Acute CGM glucose iAUC	Fixed-effect sensitivity model		Fixed effect	2	-0.635	-1.080 to -0.191	0.005	0.000	94.4%
Acute CGM glucose iAUC	Gao 2024 replacement arm: SQUAT		Random-effects HK-REML	2	-0.983	-13.437 to 11.471	0.499	1.806	94.0%
Acute CGM glucose iAUC	Fixed-effect sensitivity model		Fixed effect	2	-0.634	-1.071 to -0.197	0.004	0.000	94.0%

Outcome	Sensitivity analysis	Omitted family	Model	*k*	Hedges' g_z	95% CI	*P* value	τ^2	I^2
Acute CGM glucose iAUC	Assumed within-participant correlation *r* = 0.25		Random-effects HK-REML	2	-0.867	-11.853 to 10.119	0.499	1.392	93.0%
Acute CGM glucose iAUC	Fixed-effect sensitivity model		Fixed effect	2	-0.621	-1.046 to -0.195	0.004	0.000	93.0%
Acute CGM glucose iAUC	Assumed within-participant correlation *r* = 0.75		Random-effects HK-REML	2	-1.493	-20.498 to 17.513	0.501	4.289	95.8%
Acute CGM glucose iAUC	Fixed-effect sensitivity model		Fixed effect	2	-0.608	-1.081 to -0.134	0.012	0.000	95.8%
Plasma glucose iAUC	Fixed-effect sensitivity model		Fixed effect	4	-0.072	-0.333 to 0.189	0.590	0.000	77.9%
Plasma glucose iAUC	Assumed within-participant correlation *r* = 0.25		Random-effects HK-REML	4	-0.133	-1.027 to 0.760	0.668	0.221	73.0%
Plasma glucose iAUC	Fixed-effect sensitivity model		Fixed effect	4	-0.078	-0.337 to 0.180	0.552	0.000	73.0%
Plasma glucose iAUC	Assumed within-participant correlation *r* = 0.75		Random-effects HK-REML	4	-0.105	-1.299 to 1.089	0.798	0.471	84.8%
Plasma glucose iAUC	Fixed-effect sensitivity model		Fixed effect	4	-0.057	-0.327 to 0.212	0.676	0.000	84.8%
Plasma insulin iAUC	Fixed-effect sensitivity model		Fixed effect	3	-0.501	-0.813 to -0.189	0.002	0.000	15.3%
Plasma insulin iAUC	Assumed within-participant correlation *r* = 0.25		Random-effects HK-REML	3	-0.456	-1.160 to 0.248	0.108	0.012	7.1%
Plasma insulin iAUC	Fixed-effect sensitivity model		Fixed effect	3	-0.446	-0.753 to -0.138	0.005	0.000	7.1%
Plasma insulin iAUC	Assumed within-participant correlation *r* = 0.75		Random-effects HK-REML	3	-0.650	-1.573 to 0.273	0.094	0.068	42.4%
Plasma insulin iAUC	Fixed-effect sensitivity model		Fixed effect	3	-0.603	-0.926 to -0.280	<0.001	0.000	42.4%
Plasma glucose iAUC	Leave-one-out	Climie 2018	Random-effects HK-REML	3	0.079	-1.155 to 1.313	0.808	0.184	74.8%
Plasma glucose iAUC	Leave-one-out	Maylor 2019	Random-effects HK-REML	3	-0.339	-1.663 to 0.986	0.386	0.201	72.1%
Plasma glucose iAUC	Leave-one-out	Wanders 2021	Random-effects HK-REML	3	-0.249	-2.027 to 1.528	0.607	0.421	83.0%
Plasma glucose iAUC	Leave-one-out	Wongpipit 2021b	Random-effects HK-REML	3	-0.019	-1.803 to 1.765	0.967	0.406	79.7%
Plasma insulin iAUC	Leave-one-out	Climie 2018	Random-effects HK-REML	2	-0.450	-3.365 to 2.466	0.300	0.040	37.6%
Plasma insulin iAUC	Leave-one-out	Maylor 2019	Random-effects HK-REML	2	-0.444	-3.665 to 2.778	0.330	0.050	36.0%
Plasma insulin iAUC	Leave-one-out	Wongpipit 2021b	Random-effects HK-REML	2	-0.737	-1.122 to -0.352	0.026	0.000	0.0%

Note. The primary interpretation is based on random-effects REML models with Hartung–Knapp confidence intervals. Fixed-effect models are sensitivity analyses only. The non-null interval after omitting Wongpipit 2021b from the plasma insulin pool indicates sensitivity to study composition in a very small evidence set and should not be interpreted as confirmatory efficacy evidence.

Supplementary Table S9. Post-registration methodological refinements and transparency status

Refinement	What changed	Reason / analysis impact	Transparency status
Operational definition refined	The de novo operational definition was clarified to require intentional active movement, bout duration ≤15 min, ≥2 bouts/day, and within-day distribution; standing-only interruptions were excluded.	Applied before final eligibility decisions and de novo synthesis; intended to improve conceptual consistency across older and newer terminology.	Updated in the PROSPERO record and reported transparently in the manuscript and this supplementary file.
Full-text availability rule clarified	Reports that could not be obtained were classified as not obtained and did not enter full-text eligibility assessment.	Final PRISMA counts include 14 reports not retrieved; this rule was applied independently of effect direction.	Updated in the PROSPERO record and reported transparently in the manuscript and this supplementary file.
Acute CGM glucose iAUC added as exploratory	Acute CGM-derived glucose iAUC was analyzed separately from plasma glucose; long-term CGM outcomes remained outside the formal quantitative synthesis.	Added because compatible acute study-level data were available; explicitly labeled exploratory and subjected to sensitivity analysis. The refinement did not change review-level inclusion decisions. It affected only outcome-level allocation by permitting two otherwise eligible study families with acute CGM glucose iAUC data to enter a separate exploratory synthesis.	Updated in the PROSPERO record and reported transparently in the manuscript and this supplementary file.

Note. PROSPERO registration: CRD420261460040. The post-registration methodological refinements listed above have been updated in the PROSPERO record and are reported transparently in the manuscript and this supplementary file.

Supplementary Table S10. Test-meal composition and meal–activity timing in primary study families contributing to the acute metabolic synthesis

Study family	Setting and dietary exposure	Energy and macronutrients	Meal timing	Eligible activity timing	Outcome window
**Bailey 2022**	Four-day free-living regimen; self-selected intake recorded during the first condition and replicated during the second. Participants were asked to consume ≥3 meals/day containing ≥50% carbohydrate.	Daily mean: 9460 ± 2832 kJ; carbohydrate 294 ± 109 g (63% energy); protein 86 ± 25 g (18%); fat 86 ± 38 g (19%). A standardized pre-regimen pasta dinner contained 464 ± 2 kcal, 80.7 ± 2.8 g carbohydrate, 18.2 ± 1.2 g protein, and 7.0 ± 1.1 g fat.	Meal timing was recorded and replicated across conditions, but meals were not fixed relative to each interruption.	Standing or activity for 3–5 min at least every 30 min during ≥10 waking hours (≥20 interruptions/day); no fixed meal-to-activity delay.	24 h CGM net iAUC
**Climie 2018**	Laboratory evening meal; commercially available chicken-and-rice dinner consumed within 20 min.	Dinner provided 45% of estimated daily energy requirements; 53–55% carbohydrate, 12–15% protein, and 30–33% fat (trial meal: 5186 ± 374 kJ).	Dinner immediately preceded the 3.5 h movie/condition.	Three-minute light body-weight resistance activity during commercial breaks every 20 min; first bout approximately 20 min after the meal/condition began.	3.5 h plasma iAUC
**Gao 2024**	Controlled 8.5 h laboratory protocol with standardized breakfast and lunch.	Breakfast: 599.6 kcal; carbohydrate 57.7 g (35.5% energy), protein 23.8 g (14.5%), fat 36.1 g (50%). Lunch: 1100 kcal; carbohydrate 187.9 g (69.4%), protein 29.5 g (10.9%), fat 23.7 g (19.7%).	Breakfast at condition start, consumed in ~15 min; lunch at 3.5 h.	Three-minute walking every 45 min for 10 bouts; first bout at 1 h, approximately 45 min after breakfast completion.	8.5 h CGM net iAUC
**Gale 2023**	Controlled 4 h evening laboratory protocol; dinner at baseline and dessert at 2 h, each consumed within 15 min.	Dinner and dessert provided 34% and 10% of estimated daily energy requirements, respectively; combined composition: carbohydrate 114.8 ± 21.3 g (59% energy), protein 26.9 ± 5.0 g (15%), fat 23.4 ± 4.3 g (26%).	Dinner at baseline; dessert at 120 min.	Three-minute simple resistance activity every 30 min; first bout at 30 min, approximately 15 min after dinner completion.	4 h plasma iAUC
**Maylor 2019**	Controlled 7.5 h laboratory protocol with two standardized mixed meals.	Meal 1: 1274.4 ± 240.2 kJ; carbohydrate 43.1 ± 8.1 g (58% energy), protein 10.7 ± 2.0 g (14%), fat 9.4 ± 1.8 g (28%); predicted GI 80. Meal 2: 2123.4 ± 220.9 kJ; carbohydrate 57.6 ± 10.0 g (46%), protein 17.8 ± 3.1 g (14%), fat 22.3 ± 3.9 g (40%); predicted GI 58.	Meals at 15 and 180 min.	Primary HIGH-FREQ arm: 2 min at 65% VO2peak every 30 min; first bout at 0 min, 15 min before Meal 1.	7.5 h plasma iAUC
**Wanders 2021**	Controlled 4 h laboratory protocol; isocaloric HPLF or WEST breakfast.	HPLF: 438 kcal; carbohydrate 57.0 g (52% energy), protein 33.8 g (31%), fat 5.4 g (11%). WEST: 439 kcal; carbohydrate 49.9 g (45%), protein 15.9 g (14%), fat 19.2 g (39%).	Breakfast immediately before the 4 h sitting/activity condition.	Five-minute moderate-intensity cycling every 30 min; first bout 30 min after condition/meal start.	4 h plasma iAUC
**Wongpipit 2021b**	Controlled laboratory protocol with two identical standardized mixed meals (white bread, ham, cheese, egg, milk, and apple juice), each consumed within 10 min.	Per meal: 796 ± 57 kcal; carbohydrate 109.4 ± 7.7 g (55% energy), protein 29.8 ± 2.1 g (15%), fat 27.0 ± 1.9 g (30%).	Meals at 0 and 3 h.	Primary arm: 3 min light walking every 30 min for 10 bouts; first bout at 30 min, approximately 20 min after completion of Meal 1.	6 h plasma iAUC

Note. Timing relative to meal completion was calculated only when the original report provided a sufficiently explicit experimental timeline; approximate values are labeled accordingly. No unreported glycemic-index or nutrient values were inferred. Bailey 2022 did not prescribe a fixed meal-to-activity delay under its free-living protocol. CGM, continuous glucose monitoring; GI, glycemic index; HPLF, high-protein/low-fat meal; iAUC, incremental area under the curve; WEST, Western meal.

Supplementary Table S11. Sex composition of primary study families contributing to the acute metabolic synthesis

Study family	Study sample, *n*	Outcome-analysis *n*	Male, *n* (%)	Female, *n* (%)	Clarification
**Bailey 2022**	12	12	4 (33.3%)	8 (66.7%)	Sex composition corresponds to the analyzed sample.
**Climie 2018**	9	9	5 (55.6%)	4 (44.4%)	Sex composition corresponds to the analyzed sample.
**Gao 2024**	18	18	18 (100%)	0	Men-only study.
**Gale 2023**	30	28	8 (26.7%)	22 (73.3%)	Sex reported for the complete study sample; the sex composition of the 28 participants entering the outcome analysis was not reported separately.
**Maylor 2019**	14	14	0	14 (100%)	Women-only study.
**Wanders 2021**	24	16	5 (20.8%)	19 (79.2%)	Sex reported for the complete study sample; the sex composition of the 16 participants contributing data at the selected outcome time point was not reported separately.
**Wongpipit 2021b**	24	18	24 (100%)	0	Men-only study; the outcome-analysis subset was therefore also male.

Note. Sex composition is reported for the complete study sample unless the original report explicitly provided the composition of the outcome-analysis sample. Outcome-analysis sample sizes correspond to the estimates used in the present synthesis. Percentages may not total 100% because of rounding.

Supplementary File S1. Full electronic search strategies

PubMed/MEDLINE

```
(
 "exercise snack"[tiab]
 OR "exercise snacks"[tiab]
 OR "exercise snacking"[tiab]
 OR "movement snack"[tiab]
 OR "movement snacks"[tiab]
 OR "activity snack"[tiab]
 OR "activity snacks"[tiab]
 OR "physical activity snack"[tiab]
 OR "physical activity snacks"[tiab]
 OR snacktivity[tiab]
 OR snacktivities[tiab]
 OR "micro workout"[tiab]
 OR "micro workouts"[tiab]
 OR micro-workout*[tiab]
 OR microworkout*[tiab]
 OR "exercise burst"[tiab]
 OR "exercise bursts"[tiab]
 OR "physical activity burst"[tiab]
 OR "physical activity bursts"[tiab]
 OR "brief exercise bout"[tiab]
 OR "brief exercise bouts"[tiab]
 OR "short exercise bout"[tiab]
 OR "short exercise bouts"[tiab]
 OR "intermittent exercise bout"[tiab]
 OR "intermittent exercise bouts"[tiab]
 OR "accumulated exercise"[tiab]
 OR "vigorous intermittent lifestyle physical activity"[tiab]
 OR VILPA[tiab]
 OR "movement break"[tiab]
 OR "movement breaks"[tiab]
 OR "activity break"[tiab]
 OR "activity breaks"[tiab]
 OR "active break"[tiab]
 OR "active breaks"[tiab]
 OR "exercise break"[tiab]
 OR "exercise breaks"[tiab]
 OR "physical activity break"[tiab]
 OR "physical activity breaks"[tiab]
 OR "sedentary interruption"[tiab]
 OR "sedentary interruptions"[tiab]
```

```
  OR "interrupting prolonged sitting"[tiab]
  OR "breaking up prolonged sitting"[tiab]
  OR "breaking up sitting"[tiab]
  OR "breaking sedentary time"[tiab]
)
AND systematic[sb]
NOT (protocol[ti] OR protocols[ti])
```

Embase.com

```
(
  'exercise snack*':ti,ab,kw
  OR 'exercise snacking':ti,ab,kw
  OR 'movement snack*':ti,ab,kw
  OR 'activity snack*':ti,ab,kw
  OR 'physical activity snack*':ti,ab,kw
  OR snacktivit*:ti,ab,kw
  OR 'micro workout*':ti,ab,kw
  OR micro-workout*:ti,ab,kw
  OR microworkout*:ti,ab,kw
  OR 'exercise burst*':ti,ab,kw
  OR 'physical activity burst*':ti,ab,kw
  OR ((brief OR short OR intermittent) NEAR/3 'exercise bout*'):ti,ab,kw
  OR 'accumulated exercise':ti,ab,kw
  OR 'vigorous intermittent lifestyle physical activity':ti,ab,kw
  OR VILPA:ti,ab,kw
  OR ((movement OR activity OR active OR exercise OR 'physical activity') NEAR/2 break*):ti,ab,kw
  OR (sedentary NEAR/3 interrupt*):ti,ab,kw
  OR ((interrupt* OR break*) NEAR/4 ('prolonged sitting' OR sitting OR 'sedentary time')):ti,ab,kw
)
AND
(
  'systematic review'/de
  OR 'meta analysis'/de
  OR 'systematic review':ti,ab
  OR 'systematic reviews':ti,ab
  OR meta-analy*:ti,ab
  OR metaanaly*:ti,ab
  OR 'evidence synthesis':ti,ab
)
NOT
(
  protocol:ti
  OR protocols:ti
)
```

Web of Science Core Collection

```
TS=(
  "exercise snack*"
  OR "exercise snacking"
  OR "movement snack*"
  OR "activity snack*"
  OR "physical activity snack*"
  OR snacktivit*
  OR "micro workout*"
  OR micro-workout*
  OR microworkout*
  OR "exercise burst*"
  OR "physical activity burst*"
  OR ((brief OR short OR intermittent) NEAR/3 "exercise bout*")
  OR "accumulated exercise"
  OR "vigorous intermittent lifestyle physical activity"
  OR VILPA
```

OR ((movement OR activity OR active OR exercise OR "physical activity") NEAR/2 break*)
OR (sedentary NEAR/3 interrupt*)
OR ((interrupt* OR break*) NEAR/4 ("prolonged sitting" OR sitting OR "sedentary time"))
)
AND TS=(
"systematic review*"
OR meta-analy*
OR metaanaly*
OR "evidence synthesis"
)
NOT TI=(protocol OR protocols)

Scopus

TITLE-ABS-KEY(
"exercise snack*"
OR "exercise snacking"
OR "movement snack*"
OR "activity snack*"
OR "physical activity snack*"
OR snacktivit*
OR "micro workout*"
OR micro-workout*
OR microworkout*
OR "exercise burst*"
OR "physical activity burst*"
OR ((brief OR short OR intermittent) W/3 "exercise bout*")
OR "accumulated exercise"
OR "vigorous intermittent lifestyle physical activity"
OR VILPA
OR ((movement OR activity OR active OR exercise OR "physical activity") W/2 break*)
OR (sedentary W/3 interrupt*)
OR ((interrupt* OR break*) W/4 ("prolonged sitting" OR sitting OR "sedentary time"))
)
AND TITLE-ABS-KEY(
"systematic review*"
OR meta-analy*
OR metaanaly*
OR "evidence synthesis"
)
AND NOT TITLE(protocol OR protocols)

Cochrane Database of Systematic Reviews

#1 ("exercise snack*" OR "exercise snacking" OR "movement snack*" OR "activity snack*" OR "physical activity snack*" OR snacktivit*):ti,ab,kw

#2 ("micro workout*" OR micro-workout* OR microworkout* OR "exercise burst*" OR "physical activity burst*"):ti,ab,kw

#3 ((brief OR short OR intermittent) NEAR/3 "exercise bout*"):ti,ab,kw

#4 ("accumulated exercise" OR "vigorous intermittent lifestyle physical activity" OR VILPA):ti,ab,kw

#5 ((movement OR activity OR active OR exercise OR "physical activity") NEAR/2 break*):ti,ab,kw

#6 (sedentary NEAR/3 interrupt*):ti,ab,kw

#7 ((interrupt* OR break*) NEAR/4 ("prolonged sitting" OR sitting OR "sedentary time")):ti,ab,kw

#8 #1 OR #2 OR #3 OR #4 OR #5 OR #6 OR #7

SPORTDiscus via EBSCOhost

```
(
 TI (
  "exercise snack*" OR "exercise snacking" OR "movement snack*" OR
  "activity snack*" OR "physical activity snack*" OR snacktivit* OR
  "micro workout*" OR micro-workout* OR microworkout* OR
  "exercise burst*" OR "physical activity burst*" OR
  ((brief OR short OR intermittent) N3 "exercise bout*") OR
  "accumulated exercise" OR
  "vigorous intermittent lifestyle physical activity" OR VILPA OR
  ((movement OR activity OR active OR exercise OR "physical activity") N2 break*) OR
  (sedentary N3 interrupt*) OR
  ((interrupt* OR break*) N4 ("prolonged sitting" OR sitting OR "sedentary time"))
 )
 OR
 AB (
  "exercise snack*" OR "exercise snacking" OR "movement snack*" OR
  "activity snack*" OR "physical activity snack*" OR snacktivit* OR
  "micro workout*" OR micro-workout* OR microworkout* OR
  "exercise burst*" OR "physical activity burst*" OR
  ((brief OR short OR intermittent) N3 "exercise bout*") OR
  "accumulated exercise" OR
  "vigorous intermittent lifestyle physical activity" OR VILPA OR
  ((movement OR activity OR active OR exercise OR "physical activity") N2 break*) OR
  (sedentary N3 interrupt*) OR
  ((interrupt* OR break*) N4 ("prolonged sitting" OR sitting OR "sedentary time"))
 )
)
AND
(
 TI ("systematic review*" OR meta-analy* OR metaanaly* OR "evidence synthesis")
 OR
 AB ("systematic review*" OR meta-analy* OR metaanaly* OR "evidence synthesis")
)
NOT TI (protocol OR protocols)
```

Europe PMC

(

TITLE_ABS:"exercise snack"

OR TITLE_ABS:"exercise snacks"

OR TITLE_ABS:"exercise snacking"

OR TITLE_ABS:"movement snack"

OR TITLE_ABS:"movement snacks"

OR TITLE_ABS:"activity snack"

OR TITLE_ABS:"activity snacks"

OR TITLE_ABS:"physical activity snack"

OR TITLE_ABS:"physical activity snacks"

OR TITLE_ABS:snacktivity

OR TITLE_ABS:snacktivities

OR TITLE_ABS:"micro workout"

OR TITLE_ABS:"micro workouts"

OR TITLE_ABS:"exercise burst"

OR TITLE_ABS:"exercise bursts"

OR TITLE_ABS:"physical activity burst"

OR TITLE_ABS:"physical activity bursts"

OR TITLE_ABS:"brief exercise bout"

OR TITLE_ABS:"brief exercise bouts"

OR TITLE_ABS:"short exercise bout"

OR TITLE_ABS:"short exercise bouts"

OR TITLE_ABS:"intermittent exercise bout"

OR TITLE_ABS:"intermittent exercise bouts"

OR TITLE_ABS:"accumulated exercise"

OR TITLE_ABS:"vigorous intermittent lifestyle physical activity"

OR TITLE_ABS:VILPA

OR TITLE_ABS:"movement break"

OR TITLE_ABS:"movement breaks"

OR TITLE_ABS:"activity break"

OR TITLE_ABS:"activity breaks"

OR TITLE_ABS:"active break"

OR TITLE_ABS:"active breaks"

OR TITLE_ABS:"exercise break"

OR TITLE_ABS:"exercise breaks"

OR TITLE_ABS:"physical activity break"

OR TITLE_ABS:"physical activity breaks"

OR TITLE_ABS:"sedentary interruption"

OR TITLE_ABS:"sedentary interruptions"

OR TITLE_ABS:"interrupting prolonged sitting"

OR TITLE_ABS:"breaking up prolonged sitting"

OR TITLE_ABS:"breaking up sitting"

OR TITLE_ABS:"breaking sedentary time"

)

AND

(

TITLE_ABS:"systematic review"

OR TITLE_ABS:"systematic reviews"

OR TITLE_ABS:"meta-analysis"

OR TITLE_ABS:"meta-analyses"

OR TITLE_ABS:"meta analysis"

OR TITLE_ABS:"meta analyses"

OR TITLE_ABS:"evidence synthesis"

)

NOT TITLE:protocol

OpenAlex

"exercise snacks" AND ("systematic review" OR "meta-analysis" OR "evidence synthesis")

"exercise snacking" AND ("systematic review" OR "meta-analysis" OR "evidence synthesis")

"accumulated exercise" AND ("systematic review" OR "meta-analysis" OR "evidence synthesis")

"activity breaks" AND ("systematic review" OR "meta-analysis" OR "evidence synthesis")

"physical activity breaks" AND ("systematic review" OR "meta-analysis" OR "evidence synthesis")

"interrupting prolonged sitting" AND ("systematic review" OR "meta-analysis" OR "evidence synthesis")

"breaking up prolonged sitting" AND ("systematic review" OR "meta-analysis" OR "evidence synthesis")

"sedentary interruptions" AND ("systematic review" OR "meta-analysis" OR "evidence synthesis")

"vigorous intermittent lifestyle physical activity" AND ("systematic review" OR "meta-analysis" OR "evidence synthesis")

VILPA AND ("systematic review" OR "meta-analysis" OR "evidence synthesis")

Crossref

query.bibliographic="exercise snacks systematic review"

query.bibliographic="exercise snacks meta-analysis"

query.bibliographic="exercise snacking systematic review"

query.bibliographic="exercise snacking meta-analysis"

query.bibliographic="accumulated exercise systematic review"

query.bibliographic="accumulated exercise meta-analysis"

query.bibliographic="activity breaks systematic review"

query.bibliographic="activity breaks meta-analysis"

query.bibliographic="physical activity breaks systematic review"

query.bibliographic="physical activity breaks meta-analysis"

query.bibliographic="interrupting prolonged sitting systematic review"

query.bibliographic="interrupting prolonged sitting meta-analysis"

query.bibliographic="breaking up prolonged sitting systematic review"

query.bibliographic="breaking up prolonged sitting meta-analysis"

query.bibliographic="sedentary interruptions systematic review"

query.bibliographic="sedentary interruptions meta-analysis"

query.bibliographic="vigorous intermittent lifestyle physical activity systematic review"

query.bibliographic="VILPA systematic review"
